# A model for emergency supply management under extended EDAS method and spherical hesitant fuzzy soft aggregation information

**DOI:** 10.1038/s41598-023-35390-3

**Published:** 2023-05-24

**Authors:** Shahzaib Ashraf, Muhammad Sohail, Razia Choudhary, Muhammad Naeem, Gilbert Chambashi, Mohamed R. Ali

**Affiliations:** 1 Institute of Mathematics, Khwaja Fareed University of Engineering and Information Technology, Rahim Yar Khan, 64200 Pakistan; 2 Department of Mathematics, Deanship of Applied Sciences, Umm Al-Qura University, Macca, Saudi Arabia; 3 School of Business Studies, Unicaf University, Longacres, Lusaka, Zambia; 4 Faculty of Engineering and Technology, Future University, Cario, Egypt

**Keywords:** Natural hazards, Mathematics and computing

## Abstract

Due to the frequent occurrence of numerous emergency events that have significantly damaged society and the economy, the need for emergency decision-making has been manifest recently. It assumes a controllable function when it is critical to limit property and personal catastrophes and lessen their negative consequences on the natural and social course of events. In emergency decision-making problems, the aggregation method is crucial, especially when there are more competing criteria. Based on these factors, we first introduced some basic concepts about SHFSS, and then we introduced some new aggregation operators such as the spherical hesitant fuzzy soft weighted average, spherical hesitant fuzzy soft ordered weighted average, spherical hesitant fuzzy weighted geometric aggregation, spherical hesitant fuzzy soft ordered weighted geometric aggregation, spherical hesitant fuzzy soft hybrid average, and spherical hesitant fuzzy soft hybrid geometric aggregation operator. The characteristics of these operators are also thoroughly covered. Also, an algorithm is developed within the spherical hesitant fuzzy soft environment. Furthermore, we extend our investigation to the Evaluation based on the Distance from Average Solution method in multiple attribute group decision-making with spherical hesitant fuzzy soft averaging operators. And a numerical illustration for “supply of emergency aid in post-flooding the situation” is given to show the accuracy of the mentioned work. Then a comparison between these operators and the EDAS method is also established in order to further highlight the superiority of the established work.

## Introduction

Zadeh^1^ presented the fuzzy sets to explain the uncertainty of evaluation information and offered a way to deal with the difficulties of gathering accurate data for multi-attribute decision-making confusion. The theory of fuzzy sets has developed over time and across many disciplines since its beginning in 1965. The membership grade in the fuzzy set is close to [0, 1], but in several real-world applications, we additionally deal with non-membership grades. As a result, Atanassov^2^ prolonged the theory of FS to the intuitionistic fuzzy set (IFS), which compensates for the shortcoming of FS. Many researchers have become interested in IFS and utilized it to achieve their expected outcome in the real-world structure of DMPs. Even though non-membership grade (NMG) is engaged with the membership grade (MG) under the condition of $0\le MG+NMG\le 1$, IFS enhances the context for decision-makers (DMs). The generalized intuitionistic fuzzy aggregation operators were developed by Zhao et al.^3^. In addition^4^, introduces some intuitionistic fuzzy ordered weighted average (IFOWA), intuitionistic fuzzy hybrid average aggregation operators (IFHA), and intuitionistic fuzzy weighted average (IFWA) operators. Furthermore^5^, establishes IFAO as well as IF hybrid arithmetic and geometric aggregation operators. Subsequently, Interval values were then used to distinguish between the MG and NMG, and a new concept named “interval-valued IFS” (IVIFS) was introduced by^6^ as the specialization of FS and IFS. IFS and IVIFS concepts applied to a variety of problems, including collective decision-making^7^, similarity measures^8^, and MCDM dilemmas^9^. Zhang et al.^10^ presented several content for interval-valued IFS. While in many problems, decision-makers used data in the form of $\ `0.6$’ and ‘0.5’ as MG and NMG and IFS fail to effectively manage this type of data. In order to address this situation, Yager^11^ enhanced the concept of IFS and initiated the Pythagorean fuzzy set (PyFS) under the criterion, $0\le MG^{2}+NMG^{2}\le 1$. As a matter of fact, PyFS conveys more effective information, so IFS can be perceived as a subset of PyFS. Khan et al.^12^ initiated the Pythagorean fuzzy Dombi aggregation operators and their application in DMPs, even though aggregation operators are extremely useful in transforming the total amount of data to a single number that aids us in DMPs by choosing the best option out of the available ones. Furthermore^13^, proposes PyF interaction AO and its application in MADM. In addition, Liu and Wang^14^ invented the archimedean Bonferroni operators (ABO) for multiple-attribute decision-making. Despite the fact that many decision-making situations call for us to take the neutral grade into account, none of the theories offered above can consider anything other than MG and NMG, Cuong^15^ introduced picture fuzzy set (PFS) to overcome this limitation by the addition of another grade i.e. neutral grade (nMG). Based on PFS, Cuong et al.^16^ Introduce the conjunction, disjunction, negation, and implication essential fuzzy logic operators. Wang et al.^17^ also propose some concepts and operational laws, and they discuss some other PF geometric aggregation operators and their properties. Wei^18^ and Zeng et al.^19^ also discuss some PF aggregation operators. Zeng and colleagues^20^ characterized an improved model of textual picture fuzzy topsis strategy and its use in the Oracle E-Business Suite. We also have a condition in the picture fuzzy set $0\le MG+nMG+NMG\le 1$. However, in some circumstances, the information offered by experts cannot be addressed by PFS. For example, we can see that sum $(0.6,0.5,0.3)\notin [0,1]$ when specialists offer $``0.6''$ as MG, $``0.5''$ as nMG, and $``0.3''$ as NMG. Mahmood et al.^21^ proposed a spherical fuzzy set to overcome these difficulties, with the condition that $0\le MG^{2}+nMG^{2}+NMG^{2}\le 1$. As a result, SFS is   a more generalized case, which gives decision-makers more flexibility in several MCDM dilemmas. In decision support systems, Jin et al.^22^ discovered spherical fuzzy logarithmic AO that relies on entropy. Additionally, based on the SF framework^23,24^, explored a number of weighted average, weighted geometric, and harmonic mean AO and its uses in GDM issues. Ashraf et al.^25^ also presented spherical fuzzy Dombi AO. To aggregate the information of spherical fuzzy, Ashraf et al.^26^ initiated the GRA method, which focused on a spherical linguistic fuzzy Choquet integral environment. The TOPSIS method, developed by Ali et al.^27^, depends on a complex spherical fuzzy set with such a BM operator. It should be noted that all of the preceding existing literature solely addresses fuzzy data and does not take the parameterization structure under consideration. As a result, Molodtsov^28^ proposed the idea of a “Soft Set” (SS), which is more general than the fuzzy set due to its parameterization structure. Maji et al.^29^ proposed the idea of the fuzzy soft set (FSS), with the combination of FS and SS. In addition,^30–32^ established the application areas of FSS theory to medical conditions, decision-making challenges, and BCK/BC algebra. FSS is generalized through Interval type-2 fuzzy^33^, which is a more powerful apparatus for dealing with fuzzy set theory and in decision making problems^34^. Moreover, Garg as well as Arora^35^ presented and proposed applications for Bonferroni mean arithmetic operators in an IFSS environment. Furthermore,^36^ established the idea of IF parameterized SS theory and its utilization in decision-making. Because IFSS is a limited concept, Peng et al.^37^ established the concept of Pythagorean FSS (PyFSS). Tang tackle the DMPs under R set^34^, q-rung orthopair set and the rough q-rung orthopair set^38,39^. Husain et al.^40^ define the aggregation operators of q-rung orthopair FS set, which generalizes the intuitionistic FSS along with the Pythagorean FSS and some q-rung orthopair FS aggregation operators. Because FSS, IFSS, PyFSS, and qROFSS just explore MG and NMG whereas nMG was not mentioned. Kha^41^ merged SS and PFS, to initiate a holistic concept named picture fuzzy soft set (PFSS). Jan et al.^42^ also introduced and discussed multi-valued picture FSS in GDMPs. Moreover, SFS and SS are merged to establish the new concept known as a spherical fuzzy soft set (SFSS), which is the generalization of the PFSS and is discussed in^43^. Furthermore^44,45^, introduced the idea of an interval-valued neutrosophic fuzzy soft set and a bipolar fuzzy neutrosophic fuzzy soft set, as well as its implementation in DMPs. On the other hand, another drawback of FS is that occasionally it can be challenging to determine the precise membership degree of evaluation information. Torra^46^ created the HFS in order to represent membership degrees using a variety of possible crisp numbers. HFS is the most common method of keeping DMPs ambiguous. Babitha et al.^47^ developed the most imported notion of HFSS. Rui Wang and Yanlai Li^48^ developed the novel idea of picture hesitant fuzzy set its DM method and also mentioned its application in complex MCDM in order to address the practical issues of MCDM.

In order to deal with various MADM problems, Keshavarz et al.^49^ initially proposed the EDAS method. In particular, when incompatible criteria occur in MADM problems, then the EDAS method is very effective. Some conventional distances are also derived for the EDAS method in a manner similar to the VIKOR method (Mirghafoori et al.^50^) and TOPSIS method (Liang et al.^51^) . On the basis of the average solution, the EDAS method is regarded as PDAS and NDAS (AS). The ideal alternative ought to have a major PDAS value and a minimal NDAS value (Keshavarz et al.^52^) . The EDAS method was developed by Kahraman et al.^53^ using IFSs. The EDAS method was used by Keshavarz et al.^54^ to improve a stochastic multi-attribute decision-making process. The EDAS method was established by Keshavarz et al.^55^ in a dynamic multi-attribute decision-making approach. Stevic et al.^56^ used one of the novel methods based on the fuzzy EDAS method’s multicriteria analysis to identify the best PVC carpentry manufacturer for apartment renovations. Somehow, EmDMPs are frequently a significant problem with picture-hesitant fuzzy sets.

So, we introduced a spherical hesitant fuzzy soft set, by taking into account the hesitant fuzzy sets along with the membership, neutral, and non-membership grades and with the parameterized structure. Moreover, using the aggregation operator in spherical hesitant fuzzy soft computing, we propose a novelistic approach to MCDM problems. Furthermore, we extend our investigation to the EDAS method in MADM under SHFS averaging operators. The important purpose of our study is to develop a unique approach that could be used more successfully to resolve some MADM concerns in the context of the EDAS method with SHFSS. Motivation of spherical hesitant fuzzy soft sets are developed to further enhance the flexibility and expressiveness of fuzzy soft sets by allowing the representation of uncertainty in a spherical region (MG, nMG, NMG). This approach provides a more robust and accurate representation of uncertain and vague information, making it useful in a wide range of decision-making problems, including pattern recognition, machine learning, and multi-criteria decision-making. SHFSS address all the features that must be considered during decision-making, such as parameterizations (by soft set), hesitation and psychological effects (by hesitant effect). So, it is the most effective technique for advanced challanges in MADM.

Moreover, we’ve organized our article below: The fundamental concepts of FS, SS, FSS, HSS, HFSS, PFS, PFSS, PHFSS, SFS, SFSS, and SHFSS are discussed in “Preliminaries” section. In next “Spherical hesitant fuzzy soft set and their operational laws ” section, we defined the score and accuracy function. Furthermore, the aggregation operators based on “Spherical Hesitant fuzzy soft set” and their related theorems are thoroughly discussed.  “Spherical hesitant fuzzy soft average aggregation operators” section deals with the MCDM application based on these operators. Eventually, in “Decision making model under shfs aggregation information” section, we furnished a numerical example and gave a comparative analysis by ranking our proposed work to back it up. Furthermore in “Extended EDAS methodology” section EDAS method is used to cope with the MADM challenges in SHFS averaging operators. And a numerical illustration is used to support the effectiveness of the proposed method. After that, the comparative study is mentioned for the proposed work is mentioned to show the superiority of our work. And at the end the conclusion is given to show how established the work performs better than prior articles along with its limitations and the future work .

## Preliminaries

### Definition 2.1

^1^On a nonempty set *R*,where $\vartheta (a)$ $:R\rightarrow [0,1]$ represent the membership grade (MG), the fuzzy set (FS) is furnished as

$$\begin{aligned} \acute{Z}=\{(a,\vartheta (a)):a\in R\}. \end{aligned}$$

### Definition 2.2

^28^Over the fixed universal set *R*,and *T* as the parametric set,the duo (*S*, *T*) is referred to being a soft set (*SS*) , such that $M\subseteq T,$ the power set of *R* is *P*(*R*), where the map *S* is furnished as

$$\begin{aligned} S:M\rightarrow P(R). \end{aligned}$$

### Definition 2.3

^29^Let *T* be the parametric set, *R* is the universal set, and $M\subseteq T.$ A pair (*S*, *T*) over *R*, is supposed to be fuzzy soft set (*FSS*), with the map *S* is furnished as $S:M\rightarrow$Æ, as defined by

$$\begin{aligned} S_{\rho j}(a_{i})=\{(a_{i},\vartheta _{j}(a_{i})):a_{i}\in R\} \end{aligned}$$

where Æ is the collection of all FSS on *R*. Where $\vartheta _{j}(a_{i})$ indicates the MG meets the condition that $0\le \vartheta _{j}(a_{i})\le 1$.

### Definition 2.4

^46^Over the universal set *R* the hesitant fuzzy set (*HFS*) in aspect of a function, when implemented to *R* yields a subsets of interval$\ [0,1]$,that is depicted as ;

$$\begin{aligned} \ {\hat{H}}=\{(a_{i},\vartheta _{{\hat{H}}}(a_{i}))|a_{i}\in R\} \end{aligned}$$

where $a_{i}\in R$ to the set ${\hat{H}}$, $\vartheta _{{\hat{H}}}(a_{i})$ is a collection of values in interval [0, 1], is the membership degree of the number.

### Definition 2.5

^47^Let ${\hat{h}}(a)$ is the collection of all hesitant fuzzy sets in *R* ; a pair $({\hat{H}},O)$ is termed as hesitant fuzzy soft set (*HFSS*) over *R*, with the mapping ${\hat{H}}$ is defined as;

$$\begin{aligned} {\hat{H}}:O\rightarrow {\hat{h}}(a) \end{aligned}$$

### Definition 2.6

^17^Over the universal set *R*, the picture fuzzy set (*PFS*) is given by

$$\begin{aligned} P=\{(a,(\vartheta (a),\xi (a),\partial (a))):a\in R\} \end{aligned}$$

where $\vartheta (a):R\rightarrow [0,1]$ is the MG, $\xi (a):R\rightarrow [0,1]$ is the nMG and $\partial (a)\ :R\rightarrow [0,1]$ is NMG through the premise that $0\le \vartheta (a),\xi (a),\partial (a)\le 1$.

### Definition 2.7

^48^Over the universal set *R* the picture hesitant fuzzy set (*PHFS*) is a function, when implemented to *R* yields a subsets of $\ [0,1]$,that can be depicted as;

$$\begin{aligned} \ \check{N}=\{(a,(\vartheta (a),\xi (a),\partial (a)))|a\in R\} \end{aligned}$$

where $\vartheta (a)=\{\phi |\phi \in \vartheta (a)\},\xi (a)=\{\chi |\chi \in \xi (a)\},\partial (a)=\{\psi |\psi \in \partial (a)\}$ is a collection of three sets of values in interval [0, 1], is the membership,neutral and non-membership degree of the elements. Where $a\in R$ to the set $\ \check{N}$ over the condition that $0\le \phi ^{+}+\chi ^{+}+\psi ^{+}\le 1$, where $\phi ^{+}=\cup _{\phi \in \vartheta (a)}\max \{\phi \},\chi ^{+}=\cup _{\chi \in \xi (a)}\max \{\chi \}$ and $\psi ^{+}=\cup _{\psi \in \partial (a)}\max \{\psi \}.$ And $\check{n}=\{\vartheta ,\xi ,\partial \}$ represents the PHFE.

### Definition 2.8

^41^Over the universal set R and T be a parametric set. A pair (*S*, *T*) is known as the picture fuzzy soft set (*PFSS*), with $M\subseteq T$ and the mapping S is; $S:M\rightarrow$Œ defined by

$$\begin{aligned} S_{\rho j}(a_{i})=\{(a_{i},(\vartheta _{j}(a_{i}),\xi _{j}(a_{i}),\partial _{j}(a_{i})):a_{i}\in R\} \end{aligned}$$

where Œ over R is the collection of all PFSS over R. Where $\vartheta _{j}(a_{i}),\xi _{j}(a_{i})$ and $\partial _{j}(a_{i})$ reflect the MG, nMG, and NMG that satisfy the condition $0\le \vartheta _{j}(a_{i}),\xi _{j}(a_{i}),\partial _{j}(a_{i})\le 1$.

### Definition 2.9

^23^A spherical fuzzy set over the universal set R is the form of 
where $\vartheta (a)$ $:R\rightarrow [0,1]$ is the MG, $\xi (a)$ $:R\rightarrow [0,1]$ is the nMG and $\partial (a)$ $:R\rightarrow [0,1]$ is NMG through the premise that $0\le (\vartheta (a))^{2}+(\xi (a))^{2}+(\partial (a))^{2}\le 1$.

### Definition 2.10

^57^Over the universal set *R* the spherical hesitant fuzzy set (*SHFS*) is a function, when implemented to *R* yields a subsets of [0, 1] ,that can be depicted as ;

$$\begin{aligned} \ \check{R}=\{(a,(\vartheta (a),\xi (a),\partial (a)))|a\in R\} \end{aligned}$$

where $\vartheta (a)=\{\phi ^{2}|\phi ^{2}\in \vartheta (a)\},\xi (a)=\{\chi ^{2}|\chi ^{2}\in \xi (a)\},\partial (a)=\{\psi ^{2}|\psi ^{2}\in \partial (a)\}$ is a collection of three sets of several values in [0, 1], is the membership, neutral and non-membership degree of the elements,$\ a\in R$ to the set $\check{R}$ over the condition that $0\le (\phi ^{2})^{+}+(\chi ^{2})^{+}+(\psi ^{2})^{+}\le 1$, where $(\phi ^{2})^{+}=\cup _{\phi ^{2}\in \vartheta (a)}\max \{\phi ^{2}\},(\chi ^{2})^{+}=\cup _{\chi ^{2}\in \xi (a)}\max \{\chi ^{2}\}$ and $(\psi ^{2})^{+}=\cup _{\psi ^{2}\in \partial (a)}\max \{\psi ^{2}\}.$ And $\check{r}=\{\vartheta ,\xi ,\partial \}$ represents the SHFE.

### Definition 2.11

^43^Let a universal set R and T be a parametric set. A duo (*S*, *T*) is referred to as a spherical fuzzy soft set (*SFSS*), with $M\subseteq T,m\in M$ and the mapping S is; $S:M\rightarrow \Upsilon$ defined by

$$\begin{aligned} S_{\rho j}(m)=\{(a_{i},(\vartheta _{j}(a_{i}),\xi _{j}(a_{i}),\partial _{j}(a_{i})):a_{i}\in R\} \end{aligned}$$

where $\Upsilon$ over R is the collection of all SFSS. Where $\vartheta _{j}(a_{i}),\xi _{j}(a_{i}),$ and $\partial _{j}(a_{i})$ reflect the MG, nMG, and NMG that satisfy the condition $0\le (\vartheta _{j}(a_{i}))^{2}+(\xi _{j}(a_{i}))^{2}+(\partial _{j}(a_{i}))^{2}\le 1$.

## Spherical hesitant fuzzy soft set and their operational laws

In this section, the idea of the spherical hesitant fuzzy soft set (SHFSS) and their operations and operators are introduced. It is also necessary to rank the SHFSS when applying them to practical MCDM problems; thus, we define several fundamental operationg laws for SHFSNs, score functions, and accuracy functions to aid in the selection of the most effective alternative in MCDM problems.

### Definition 3.1

Let a universal set R and T be a parametric set. A duo (*S*, *T*) is referred to as a spherical hesitant fuzzy soft set (*SHFSS*), with $M\subseteq T,m\in M$ and the mapping S is;$S:M\rightarrow \Upsilon$ defined by

$$\begin{aligned} S_{\rho ij}(m)=\{(a_{i},(\vartheta _{j}(a_{i}),\xi _{j}(a_{i}),\partial _{j}(a_{i})):a_{i}\in R\} \end{aligned}$$

where $\Upsilon$ over R is the collection of all SHFSS. With $\vartheta _{j}(a_{i})=\{\phi _{j}|\phi _{j}\in \vartheta _{j}(a_{i})\},\xi _{j}(a_{i})=\{\chi _{j}|\chi _{j}\in \xi _{j}(a_{i})\},\partial _{j}(a_{i})=\{\psi _{j}|\psi _{j}\in \partial _{j}(a_{i}))\}$ is a collection of three hesitant sets of several values in [0, 1], is the membership, neutral and non-membership degree of the elements, over the condition $\ 0\le (\phi _{j}^{+})^{2}+(\chi _{j}^{+})^{2}+(\psi _{j}^{+})^{2}\le 1$, where $(\phi ^{+})=\cup _{\phi _{j}\in \vartheta _{j}(a_{i})}\max \{\alpha _{j}\},(\chi ^{+})=\cup _{\chi _{j}\in \xi _{j}(a_{i})}\max \{\chi _{j}\}$ and $(\psi ^{+})=\cup _{\psi _{j}\in \partial _{j}(a_{i})}\max \{\psi _{j}\}.$ And ${\hat{S}}=\{\vartheta _{j}(a_{i}),\xi _{j}(a_{i}),\partial _{j}(a_{i})\}$ represents the SHFSE. 

### Definition 3.2

Let $S_{\rho rs}=\{\vartheta _{s}(a_{r}),\xi _{s}(a_{r}),\partial _{s}(a_{r})\},S_{\rho rt}=\{\vartheta _{t}(a_{r}),\xi _{t}(a_{r}),\partial _{t}(a_{r})\}$ be two SHFSNs and $\kappa >0.$ Then basic operational laws for SHFSNs are defined by

1. $$S_{\rho rs}^{c}=\cup _{\phi _{s}\in \vartheta _{s},\chi _{s}\in \xi _{s},\psi _{s}\in \partial _{s}}(\psi _{s},\chi _{s},\phi _{s}).$$
2. $$\kappa S_{\rho rs}=\cup _{\phi _{s}\in \vartheta _{s},\chi _{s}\in \xi _{s},\psi _{s}\in \partial _{s}}(\sqrt{1-(1-(\phi _{s})^{2})^{\kappa }} ,(\chi _{s})^{\kappa },(\psi _{s})^{\kappa }).$$
3. $$S_{\rho rs}^{\kappa }=\cup _{\phi _{s}\in \vartheta _{s},\chi _{s}\in \xi _{s},\psi _{s}\in \partial _{s}}(\phi _{s})^{\kappa },(\chi _{s})^{\kappa },\sqrt{1-(1-(\psi _{s})^{2})^{\kappa }}.$$
4. $$S_{\rho rs}\oplus S_{\rho rt}=\cup _{\phi _{s}\in \vartheta _{s},\chi _{s}\in \xi _{s},\psi _{s}\in \partial _{s},\phi _{t}\in \vartheta _{t},\chi _{t}\in \xi _{t},\psi _{t}\in \partial _{t}}(\sqrt{(\phi _{s})^{2}+(\phi _{t})^{2}-(\phi _{s})(\phi _{t})},(\chi _{s})(\chi _{t}),(\psi _{s})(\psi _{t})).$$
5. $$S_{\rho rs}\otimes S_{\rho rt}=\cup _{\phi _{s}\in \vartheta _{s},\chi _{s}\in \xi _{s},\psi _{s}\in \partial _{s},\phi _{t}\in \vartheta _{t},\chi _{t}\in \xi _{t},\psi _{t}\in \partial _{t}}((\phi _{s})(\phi _{t}),(\chi _{s})(\chi _{t}),\sqrt{(\psi _{s})^{2}+(\psi _{t})^{2}-(\psi _{s})^{2})(\psi _{t})^{2}}).$$

### Definition 3.3

let ${\hat{\jmath }}=$ $\{\vartheta _{s}(a_{r}),\xi _{s}(a_{r}),\partial _{s}(a_{r})\}$ be a SHFSE, the numbers of elements in $\vartheta _{s},\xi _{s},\partial _{s}$ are *x*, *y*, *z* respectively.Thus, the score function is defined as

$$\begin{aligned} sc({\hat{\jmath }})=\frac{\left( 1+\frac{1}{x}\sum \limits _{s=1}^{x}\phi _{s}- \frac{1}{y}\sum \limits _{s=1}^{y}\chi _{s}-\frac{1}{z}\sum \limits _{s=1}^{z} \psi _{s}\right) }{2},sc({\hat{\jmath }})\in [0,1]. \end{aligned}$$

and the accuracy function is;

$$\begin{aligned} ac({\hat{\jmath }})=\left( \frac{1}{x}\sum \limits _{s=1}^{x}\phi _{s}-\frac{1}{z} \sum \limits _{s=1}^{z}\psi _{s}\right) ,ac({\hat{\jmath }})\in [0,1]. \end{aligned}$$

## Spherical hesitant fuzzy soft average aggregation operators

Operators are necessary to develop a robust framework for decision-making in a spherical hesitant fuzzy soft environment, where uncertainty and hesitancy are inherent in the data. So, some of the averaging aggregated operators of SHFSS are given as:

### Spherical hesitant fuzzy soft weighted average (SHFSWA) aggregation operators

This operator is used to calculate the weighted average of a set of SHFSS, where the weights are represented by SHFSS. This operator is essential because it allows for the aggregation of multiple SHFSS with different degrees of uncertainty, which is a common scenario in decision-making problems.

#### Definition 4.1

Suppose $\Gamma (j_{r})=(\phi _{\Gamma (j_{r})},\chi _{\Gamma (j_{r})},\psi _{\Gamma (j_{r})}),(r=1,2,3,..,p)$ be collecction of SHFSNs $(\Gamma ,J)$ having WVs $\acute{r}$ $=(\acute{r}_{1},\acute{r}_{2},\acute{r} _{3},..,\acute{r}_{p})^{T}$ for $\Gamma (j_{r})$ parameters (attributes), where $\acute{r}_{r}\in [0,1]$ with $\Sigma _{r=1}^{p}\acute{r}_{r}=1$ and $\acute{r}_{r}\ge 0,$ then SHFSWA operator is the mapping defined as $SHFSWA:\Upsilon ^{p}\rightarrow \Upsilon$, where ($\Upsilon$ is the family of all SHFSNs)such that SHFSWA $\Gamma (j_{r})=(\phi _{\Gamma (j_{r})},\chi _{\Gamma (j_{r})},\psi _{\Gamma (j_{r})}),(r=1,2,3,..,p).$

$$\begin{aligned} SHFSWA(\Gamma (j_{1}),\Gamma (j_{2}),\Gamma (j_{3}),....,\Gamma (j_{p}))=\oplus _{r=1}^{p}\acute{r}_{r}\Gamma (j_{r}). \end{aligned}$$

#### Theorem 4.2

Let $\Gamma (j_{r})=(\phi _{\Gamma (j_{r})},\chi _{\Gamma (j_{r})},\psi _{\Gamma (j_{r})}),(r=1,2,3,..,p),$ be an SHFSNs, the aggregated data by SHFSWA operator is also an SHFSNs, and given by

$$\begin{aligned}{} & {} SHFSWA(\Gamma (j_{1}),\Gamma (j_{2}),\Gamma (j_{3}),....,\Gamma (j_{p})) \\= & {} \oplus _{r=1}^{p}\acute{r}_{r}\Gamma (j_{r}). \\= & {} \bigcup \limits _{\begin{array}{c} \phi _{\Gamma (j_{1})}\in \vartheta _{\Gamma (j_{1})},\phi _{\Gamma (j_{2})}\in \vartheta _{\Gamma (j_{2})},...,\phi _{\Gamma (j_{p})}\in \vartheta _{\Gamma (j_{p})}, \\ \chi _{\Gamma (j_{1})}\in \xi _{\Gamma (j_{1})},\chi _{\Gamma (j_{2})}\in \xi _{\Gamma (j_{2})},...,\chi _{\Gamma (j_{p})}\in \xi _{\Gamma (j_{p})}, \\ \psi _{\Gamma (j_{1})}\in \partial _{\Gamma (j_{1})},\psi _{\Gamma (j_{2})}\in \partial _{\Gamma (j_{2})},...,\psi _{_{\Gamma (j_{p})}}\in \partial _{_{\Gamma (j_{p})}.} \end{array}}\left\{ \begin{array}{c} \sqrt{1-\Pi _{r=1}^{p}(1-\phi _{\Gamma (j_{r})}^{2})^{\acute{r}_{r}}}, \\ \Pi _{r=1}^{p}(\chi _{\Gamma (j_{r})})^{\acute{r}_{r}},\Pi _{r=1}^{p}(\psi _{\Gamma (j_{r})})^{\acute{r}_{r}} \end{array} \right\} , \end{aligned}$$

where $r=1,2,\ldots ,p$, if $\acute{r}$ $=(\acute{r}_{1},\acute{r}_{2}, \acute{r}_{3},..,\acute{r}_{p})^{T}$ denote the WV of $\acute{r}_{r}$ parameters with condition $\acute{r}_{r}\in [0,1]$ with $\Sigma _{r=1}^{p}\acute{r}_{r}=1$ and $\acute{r}_{r}\ge 0.$

#### Proof

This conclusion has to be supported by mathematical induction.

For $$r=2$$;

$$\begin{aligned} SHFSWA(\Gamma (j_{1}),\Gamma (j_{2}))=\oplus _{k=1}^{2}\acute{r}_{r}\Gamma (j_{r})=\acute{r}_{1}\Gamma (j_{1})\oplus \acute{r}_{2}\Gamma (j_{2}). \end{aligned}$$

By using the operational law, we have:

$$\begin{aligned} \acute{r}_{1}\Gamma (j_{1})= \bigcup \limits _{\phi _{\Gamma (j_{1})}\in \vartheta _{\Gamma (j_{1})},\chi _{\Gamma (j_{1})}\in \xi _{\Gamma (j_{1})},\psi _{\Gamma (j_{1})}\in \partial _{\Gamma (j_{1})}.}\left\{ \sqrt{1-(1-\phi _{\Gamma (j_{1})}^{2})^{ \acute{r}_{1}}},(\chi _{\Gamma (j_{1})})^{\acute{r}_{1}},(\psi _{\Gamma (j_{1})})^{\acute{r}_{1}}\right\} . \end{aligned}$$

$$\begin{aligned} \acute{r}_{2}\Gamma (j_{2})= \bigcup \limits _{\phi _{\Gamma (j_{2})}\in \vartheta _{\Gamma (j_{2})},\chi _{\Gamma (j_{2})}\in \xi _{\Gamma (j_{2})},\psi _{\Gamma (j_{2})}\in \partial _{\Gamma (j_{2})}.}\left\{ \sqrt{1-(1-\phi _{\Gamma (j_{2})}^{2})^{ \acute{r}_{2}}},(\chi _{\Gamma (j_{2})})^{\acute{r}_{2}},(\psi _{\Gamma (j_{2})})^{\acute{r}_{2}}\right\} . \\ \acute{r}_{1}\Gamma (j_{1})\oplus \acute{r}_{2}\Gamma (j_{2})= \bigcup \limits _{\begin{array}{c} \phi _{\Gamma (j_{1})}\in \vartheta _{\Gamma (j_{1})},\phi _{\Gamma (j_{2})}\in \vartheta _{\Gamma (j_{2})}, \\ \chi _{\Gamma (j_{1})}\in \xi _{\Gamma (j_{1})},\chi _{\Gamma (j_{2})}\in \xi _{\Gamma (j_{2})}, \\ \psi _{\Gamma (j_{1})}\in \partial _{\Gamma (j_{1})},\psi _{\Gamma (j_{2})}\in \partial _{\Gamma (j_{2})}. \end{array}}\left\{ \begin{array}{c} \sqrt{1-(1-\phi _{\Gamma (j_{1})}^{2})^{\acute{r}_{1}}},(\chi _{\Gamma (j_{1})})^{\acute{r}_{1}},(\psi _{\Gamma (j_{1})})^{\acute{r}_{1}}\oplus \\ \sqrt{1-(1-\phi _{\Gamma (j_{2})}^{2})^{\acute{r}_{2}}},(\chi _{\Gamma (j_{2})})^{\acute{r}_{2}},(\psi _{\Gamma (j_{2})})^{\acute{r}_{2}} \end{array} \right\} . \\ \acute{r}_{1}\Gamma (j_{1})\oplus \acute{r}_{2}\Gamma (j_{2})= \bigcup \limits _{\begin{array}{c} \phi _{\Gamma (j_{1})}\in \vartheta _{\Gamma (j_{1})},\phi _{\Gamma (j_{2})}\in \vartheta _{\Gamma (j_{2})}, \\ \chi _{\Gamma (j_{1})}\in \xi _{\Gamma (j_{1})},\chi _{\Gamma (j_{2})}\in \xi _{\Gamma (j_{2})}, \\ \psi _{\Gamma (j_{1})}\in \partial _{\Gamma (j_{1})},\psi _{\Gamma (j_{2})}\in \partial _{\Gamma (j_{2})}. \end{array}}\left\{ \begin{array}{c} \sqrt{ \begin{array}{c} (1-(1-\phi _{\Gamma (j_{1})}^{2})^{\acute{r}_{1}})+(1-(1-\phi _{\Gamma (j_{2})}^{2})^{\acute{r}_{2}})- \\ (1-(1-\phi _{\Gamma (j_{1})}^{2})^{\acute{r}_{1}})(1-(1-\phi _{\Gamma (j_{2})}^{2})^{\acute{r}_{2}}) \end{array} }, \\ (\chi _{\Gamma (j_{1})})^{\acute{r}_{1}}(\chi _{\Gamma (j_{2})})^{\acute{r} _{2}},(\psi _{\Gamma (j_{1})})^{\acute{r}_{1}}(\psi _{\Gamma (j_{2})})^{ \acute{r}_{2}} \end{array} \right\} . \end{aligned}$$

$$\begin{aligned} \acute{r}_{1}\Gamma (j_{1})\oplus \acute{r}_{2}\Gamma (j_{2})= \bigcup \limits _{\begin{array}{c} \phi _{\Gamma (j_{1})}\in \vartheta _{\Gamma (j_{1})},\phi _{\Gamma (j_{2})}\in \vartheta _{\Gamma (j_{2})}, \\ \chi _{\Gamma (j_{1})}\in \xi _{\Gamma (j_{1})},\chi _{\Gamma (j_{2})}\in \xi _{\Gamma (j_{2})}, \\ \psi _{\Gamma (j_{1})}\in \partial _{\Gamma (j_{1})},\psi _{\Gamma (j_{2})}\in \partial _{\Gamma (j_{2})}. \end{array}}\left\{ \begin{array}{c} \sqrt{1-(1-\phi _{\Gamma (j_{1})}^{2})^{\acute{r}_{1}}(1-\phi _{\Gamma (j_{2})}^{2})^{\acute{r}_{2}}}, \\ (\chi _{\Gamma (j_{1})})^{\acute{r}_{1}}(\chi _{\Gamma (j_{2})})^{\acute{r} _{2}},(\psi _{\Gamma (j_{1})})^{\acute{r}_{1}}(\psi _{\Gamma (j_{2})})^{ \acute{r}_{2}} \end{array} \right\} . \\ \acute{r}_{1}\Gamma (j_{1})\oplus \acute{r}_{2}\Gamma (j_{2})= \bigcup \limits _{\begin{array}{c} \phi _{\Gamma (j_{1})}\in \vartheta _{\Gamma (j_{1})},\phi _{\Gamma (j_{2})}\in \vartheta _{\Gamma (j_{2})}, \\ \chi _{\Gamma (j_{1})}\in \xi _{\Gamma (j_{1})},\chi _{\Gamma (j_{2})}\in \xi _{\Gamma (j_{2})}, \\ \psi _{\Gamma (j_{1})}\in \partial _{\Gamma (j_{1})},\psi _{\Gamma (j_{2})}\in \partial _{\Gamma (j_{2})}. \end{array}}\left\{ \begin{array}{c} \sqrt{1-\Pi _{r=1}^{2}(1-\phi _{\Gamma (j_{r})}^{2})^{\acute{r}_{r}}}, \\ \Pi _{r=1}^{2}(\chi _{\Gamma (j_{r})})^{\acute{r}_{r}},\Pi _{r=1}^{2}(\psi _{\Gamma (j_{r})})^{\acute{r}_{r}} \end{array} \right\} . \end{aligned}$$

Thus, the results are true for $r=2$. Assume the results also hold for $p=z.$

$$\begin{aligned}{} & {} SHFSWA(\Gamma (j_{1}),\Gamma (j_{2}),\Gamma (j_{3}),....,\Gamma (j_{z})) \\= & {} \oplus _{r=1}^{z}\acute{r}_{r}\Gamma (j_{r}) \\= & {} \bigcup \limits _{\begin{array}{c} \phi _{\Gamma (j_{1})}\in \vartheta _{\Gamma (j_{1})},\phi _{\Gamma (j_{2})}\in \vartheta _{\Gamma (j_{2})},...,\phi _{\Gamma (j_{z})}\in \vartheta _{\Gamma (j_{z})}, \\ \chi _{\Gamma (j_{1})}\in \xi _{\Gamma (j_{1})},\chi _{\Gamma (j_{2})}\in \xi _{\Gamma (j_{2})},...,\chi _{\Gamma (j_{z})}\in \xi _{\Gamma (j_{z})}, \\ \psi _{\Gamma (j_{1})}\in \partial _{\Gamma (j_{1})},\psi _{\Gamma (j_{2})}\in \partial _{\Gamma (j_{2})},...,\psi _{_{\Gamma (j_{z})}}\in \partial _{_{\Gamma (j_{z})}.} \end{array}}\left\{ \sqrt{1-\Pi _{r=1}^{z}(1-\phi _{\Gamma (j_{r})}^{2})^{\acute{r}_{r}}},\Pi _{r=1}^{z}(\chi _{\Gamma (j_{r})})^{ \acute{r}_{r}},\Pi _{r=1}^{z}(\psi _{\Gamma (j_{r})})^{\acute{r}_{r}}\right\} \end{aligned}$$

Further, suppose that the results are true for $\ r=z+1,$ so combined the above two conditions, we have the following form;

$$\begin{aligned}{} & {} SHFSWA(\Gamma (j_{1}),\Gamma (j_{2}),...,\Gamma (j_{z}),\Gamma (j_{z+1})) \\= & {} \oplus _{r=1}^{z}\acute{r}_{r}\Gamma (j_{r})+\acute{r}_{z+1}\Gamma (j_{z+1}) \\= & {} \bigcup \limits _{\begin{array}{c} \phi _{\Gamma (j_{1})}\in \vartheta _{\Gamma (j_{1})},\phi _{\Gamma (j_{2})}\in \vartheta _{\Gamma (j_{2})},..., \\ \phi _{\Gamma (j_{z})}\in \vartheta _{\Gamma (j_{z})},\phi _{\Gamma (j_{z+1})}\in \vartheta _{\Gamma (j_{z+1})} \\ \chi _{\Gamma (j_{1})}\in \xi _{\Gamma (j_{1})},\chi _{\Gamma (j_{2})}\in \xi _{\Gamma (j_{2})},..., \\ \chi _{\Gamma (j_{z})}\in \xi _{\Gamma (j_{z})},\chi _{\Gamma (j_{z+1})}\in \xi _{\Gamma (j_{z+1})} \\ \psi _{\Gamma (j_{1})}\in \partial _{\Gamma (j_{1})},\psi _{\Gamma (j_{2})}\in \partial _{\Gamma (j_{2})},..., \\ \psi _{_{\Gamma (j_{z})}}\in \partial _{_{\Gamma (j_{z})}},\psi _{_{\Gamma (j_{z+1})}}\in \partial _{_{\Gamma (j_{z+1})}} \end{array}}\left\{ \begin{array}{c} \sqrt{1-\Pi _{r=1}^{z}(1-\phi _{\Gamma (j_{r})}^{2})^{\acute{r}_{r}}},\Pi _{r=1}^{z}(\chi _{\Gamma (j_{r})})^{\acute{r}_{r}},\Pi _{r=1}^{z}(\psi _{\Gamma (j_{r})})^{\acute{r}_{r}}\oplus \\ \sqrt{1-(1-\phi _{\Gamma (j_{z+1})}^{2})^{\acute{r}_{z+1}}},(\chi _{\Gamma (j_{\acute{r}_{z+1}})})^{\acute{r}_{z+1}},(\psi _{\Gamma (j_{\acute{r} _{z+1}})})^{\acute{r}_{z+1}} \end{array} \right\} \end{aligned}$$

$$\begin{aligned} =\bigcup \limits _{\begin{array}{c} \phi _{\Gamma (j_{1})}\in \vartheta _{\Gamma (j_{1})},\phi _{\Gamma (j_{2})}\in \vartheta _{\Gamma (j_{2})},..., \\ \phi _{\Gamma (j_{z})}\in \vartheta _{\Gamma (j_{z})},\phi _{\Gamma (j_{z+1})}\in \vartheta _{\Gamma (j_{z+1})} \\ \chi _{\Gamma (j_{1})}\in \xi _{\Gamma (j_{1})},\chi _{\Gamma (j_{2})}\in \xi _{\Gamma (j_{2})},..., \\ \chi _{\Gamma (j_{z})}\in \xi _{\Gamma (j_{z})},\chi _{\Gamma (j_{z+1})}\in \xi _{\Gamma (j_{z+1})} \\ \psi _{\Gamma (j_{1})}\in \partial _{\Gamma (j_{1})},\psi _{\Gamma (j_{2})}\in \partial _{\Gamma (j_{2})},..., \\ \psi _{_{\Gamma (j_{z})}}\in \partial _{_{\Gamma (j_{z})}},\psi _{_{\Gamma (j_{z+1})}}\in \partial _{_{\Gamma (j_{z+1})}} \end{array}}\left\{ \begin{array}{c} \sqrt{ \begin{array}{c} (1-\Pi _{r=1}^{z}(1-\phi _{\Gamma (j_{r})}^{2})^{\acute{r}_{r}})+(1-(1-\phi _{\Gamma (j_{z+1})}^{2})^{\acute{r}_{z+1}})- \\ (1-\Pi _{r=1}^{z}(1-\phi _{\Gamma (j_{r})}^{2})^{\acute{r}_{r}})(1-(1-\phi _{\Gamma (j_{z+1})}^{2})^{\acute{r}_{z+1}}) \end{array} }, \\ \Pi _{r=1}^{z}(\chi _{\Gamma (j_{r})})^{\acute{r}_{r}}(\chi _{\Gamma (j_{z+1})})^{\acute{r}_{z+1}},\Pi _{r=1}^{z}(\psi _{\Gamma (j_{r})})^{\acute{ r}_{r}}(\psi _{\Gamma (j_{z+1})})^{\acute{r}_{z+1}} \end{array} \right\} \end{aligned}$$

$$\begin{aligned} =\bigcup \limits _{\begin{array}{c} \phi _{\Gamma (j_{1})}\in \vartheta _{\Gamma (j_{1})},\phi _{\Gamma (j_{2})}\in \vartheta _{\Gamma (j_{2})},..., \\ \phi _{\Gamma (j_{z})}\in \vartheta _{\Gamma (j_{z})},\phi _{\Gamma (j_{z+1})}\in \vartheta _{\Gamma (j_{z+1})} \\ \chi _{\Gamma (j_{1})}\in \xi _{\Gamma (j_{1})},\chi _{\Gamma (j_{2})}\in \xi _{\Gamma (j_{2})},..., \\ \chi _{\Gamma (j_{z})}\in \xi _{\Gamma (j_{z})},\chi _{\Gamma (j_{z+1})}\in \xi _{\Gamma (j_{z+1})} \\ \psi _{\Gamma (j_{1})}\in \partial _{\Gamma (j_{1})},\psi _{\Gamma (j_{2})}\in \partial _{\Gamma (j_{2})},..., \\ \psi _{_{\Gamma (j_{z})}}\in \partial _{_{\Gamma (j_{z})}},\psi _{_{\Gamma (j_{z+1})}}\in \partial _{_{\Gamma (j_{z+1})}} \end{array}}\left\{ \begin{array}{c} \sqrt{(1-\Pi _{r=1}^{z}(1-\phi _{\Gamma (j_{r})}^{2})^{\acute{r}_{r}}(1-\phi _{\Gamma (j_{z+1})}^{2})^{\acute{r}_{z+1}}}, \\ \Pi _{r=1}^{z}(\chi _{\Gamma (j_{r})})^{\acute{r}_{r}}(\chi _{\Gamma (j_{z+1})})^{\acute{r}_{z+1}},\Pi _{r=1}^{z}(\psi _{\Gamma (j_{r})})^{\acute{ r}_{r}}(\psi _{\Gamma (j_{z+1})})^{\acute{r}_{z+1}} \end{array} \right\} \end{aligned}$$

$$\begin{aligned} =\bigcup \limits _{\begin{array}{c} \phi _{\Gamma (j_{1})}\in \vartheta _{\Gamma (j_{1})},\phi _{\Gamma (j_{2})}\in \vartheta _{\Gamma (j_{2})},...,\phi _{\Gamma (j_{z})}\in \vartheta _{\Gamma (j_{z})},\phi _{\Gamma (j_{z+1})}\in \vartheta _{\Gamma (j_{z+1})} \\ \chi _{\Gamma (j_{1})}\in \xi _{\Gamma (j_{1})},\chi _{\Gamma (j_{2})}\in \xi _{\Gamma (j_{2})},...,\chi _{\Gamma (j_{z})}\in \xi _{\Gamma (j_{z})},\chi _{\Gamma (j_{z+1})}\in \xi _{\Gamma (j_{z+1})} \\ \psi _{\Gamma (j_{1})}\in \partial _{\Gamma (j_{1})},\psi _{\Gamma (j_{2})}\in \partial _{\Gamma (j_{2})},...,\psi _{_{\Gamma (j_{z})}}\in \partial _{_{\Gamma (j_{z})}},\psi _{_{\Gamma (j_{z+1})}}\in \partial _{_{\Gamma (j_{z+1})}} \end{array}}\left\{ \begin{array}{c} \sqrt{(1-\Pi _{r=1}^{z+1}(1-\phi _{\Gamma (j_{r})}^{2})^{\acute{r}_{r}}}, \\ \Pi _{r=1}^{z+1}(\chi _{\Gamma (j_{r})})^{\acute{r}_{r}},\Pi _{r=1}^{z+1}(\psi _{\Gamma (j_{r})})^{\acute{r}_{r}} \end{array} \right\} \end{aligned}$$

It is obvious from the expression above that aggregated value is also SHFSN. Consequently, the outcome is valid for all n.

$$\begin{aligned} SHFSWA(\Gamma (j_{1}),...,\Gamma (j_{p}))=\bigcup \limits _{\begin{array}{c} \phi _{\Gamma (j_{1})}\in \vartheta _{\Gamma (j_{1})},\phi _{\Gamma (j_{2})}\in \vartheta _{\Gamma (j_{2})},...,\phi _{\Gamma (j_{p})}\in \vartheta _{\Gamma (j_{p})}, \\ \chi _{\Gamma (j_{1})}\in \xi _{\Gamma (j_{1})},\chi _{\Gamma (j_{2})}\in \xi _{\Gamma (j_{2})},...,\chi _{\Gamma (j_{p})}\in \xi _{\Gamma (j_{p})}, \\ \psi _{\Gamma (j_{1})}\in \partial _{\Gamma (j_{1})},\psi _{\Gamma (j_{2})}\in \partial _{\Gamma (j_{2})},...,\psi _{_{\Gamma (j_{p})}}\in \partial _{_{\Gamma (j_{p})}.} \end{array}}\left\{ \begin{array}{c} \sqrt{1-\Pi _{r=1}^{p}(1-\phi _{\Gamma (j_{r})}^{2})^{\acute{r}_{r}}}, \\ \Pi _{r=1}^{p}(\chi _{\Gamma (j_{r})})^{\acute{r}_{r}},\Pi _{r=1}^{p}(\psi _{\Gamma (j_{r})})^{\acute{r}_{r}} \end{array} \right\} \end{aligned}$$

which presents the proof. $\square$

#### Property 4.3

Obviously, there are some properties which are usually achieved by SHFSWA aggregation operators.

(a) Idempotency: let $\Gamma (j_{r})=(\phi _{\Gamma (j_{r})},\chi _{\Gamma (j_{r})},\psi _{\Gamma (j_{r})}),(r=1,2,3,..,p)$ be any collection of SHFSS. If all of the $\Gamma (j_{r})=(\phi _{\Gamma (j_{r})},\chi _{\Gamma (j_{r})},\psi _{\Gamma (j_{r})})$ are identical then there is:

$$\begin{aligned} SHFSWA(\Gamma (j_{1}),\Gamma (j_{2}),\Gamma (j_{3}),....,\Gamma (j_{p}))=\Gamma (j) \end{aligned}$$

(b) Monotonicity: let $\Gamma ^{\prime }(j_{r})=(\phi _{\Gamma ^{\prime }(j_{r})},\chi _{\Gamma ^{\prime }(j_{r})},\psi _{\Gamma ^{\prime }(j_{r})}),and$ $\Gamma ^{\prime }(j_{r})=(\phi _{\Gamma ^{\prime }(j_{r})},\chi _{\Gamma ^{\prime }(j_{r})},\psi _{\Gamma ^{\prime }(j_{r})})$ where $(r=1,2,3,..,p)$ be any collection of SHFSS. If it satisfied that $\Gamma (j_{r})\le$ $\Gamma ^{\prime }(j_{r})$ for whole $r\in \Upsilon$ then:

$$\begin{aligned} SHFSWA(\Gamma (j_{1}),\Gamma (j_{2}),\Gamma (j_{3}),....,\Gamma (j_{p}))=SHFSWA(\Gamma ^{\prime }(j_{1}),\Gamma ^{\prime }(j_{2}),\Gamma ^{\prime }(j_{3}),....,\Gamma ^{\prime }(j_{p})) \end{aligned}$$

(c) Boundedness: let $\Gamma (j_{r})=(\phi _{\Gamma (j_{r})},\chi _{\Gamma (j_{r})},\psi _{\Gamma (j_{r})}),(r=1,2,3,..,p)$ be any collection of SHFSS. Assuming that $\Gamma (j_{r})^{-}=(\min ^{\phi _{\Gamma (j_{r})}},\min ^{\chi _{\Gamma (j_{r})}},\max ^{\psi _{\Gamma (j_{r})}})$ and $\Gamma (j_{r})^{+}=(\max ^{\phi _{\Gamma (j_{r})}},\min ^{\chi _{\Gamma (j_{r})}},\min ^{\psi _{\Gamma (j_{r})}})$ then:

$$\begin{aligned} min\Gamma (j_{r})\le SHFSWA(\Gamma (j_{1}),\Gamma (j_{2}),\Gamma (j_{3}),....,\Gamma (j_{p}))\le \max \Gamma (j_{r}) \end{aligned}$$

### Spherical hesitant fuzzy soft ordered weighted average (SHFSOWA) operator

Since it is evident from the analysis above that SHFSWA cannot balance the order position by scoring the SHFS values, we will address the idea of a SHFSOWA operator in this part in order to get around this limitation. By grading the SHFSNs, this operator can weigh the order position. Additionally, the features of well-known operators are examined.

#### Definition 4.4

Suppose $\Gamma (j_{r})=(\phi _{\Gamma (j_{r})},\chi _{\Gamma (j_{r})},\psi _{\Gamma (j_{r})}),(r=1,2,3,..,p)$ be collecction of SHFSNs $(\Gamma ,J)$ with $\acute{r}=(\acute{r}_{1},\acute{r}_{2},\acute{r}_{3},.., \acute{r}_{p})^{T}$ WV for$\ \Gamma (j_{r})$ parameters, where $\acute{r} _{r}\in [0,1]$ with $\Sigma _{r=1}^{p}\acute{r}_{r}=1$ and $\acute{r} _{r}\ge 0,$ where $(\sigma _{1},\sigma _{2},...,\sigma _{p})$ is the variation of $\ (1,2,3,...,p)$ such that $m_{\sigma (r-1)}\ge m_{\sigma (r)}$ for all $r=2,3,...,p$. SHFSOWA operator is the mapping defined as $SHFSOWA:\Upsilon ^{p}\rightarrow \Upsilon$, where ($\Upsilon$ is the family of all SHFSNs) such that SHFSOWA $\Gamma (j_{\sigma r})=(\phi _{\Gamma (j_{\sigma r})},\chi _{\Gamma (j_{\sigma r})},\psi _{\Gamma (j_{\sigma r})}),(r=1,2,3,..,p).$

$$\begin{aligned} SHFSOWA(\Gamma (j_{\sigma 1}),\Gamma (j_{\sigma 2}),\Gamma (j_{\sigma 3}),....,\Gamma (j_{\sigma p}))=\oplus _{r=1}^{p}\acute{r}_{r}\Gamma (j_{\sigma r}). \end{aligned}$$

#### Theorem 4.5

Let $\Gamma (j_{\sigma r})=(\phi _{\Gamma (j_{\sigma r})},\chi _{\Gamma (j_{\sigma r})},\psi _{\Gamma (j_{\sigma r})}),(r=1,2,3,..,p),$ be an SHFSNs, the aggregated value by SHFSOWA operator is also an SHFSNs, and given by

$$\begin{aligned}{} & {} SHFSOWA(\Gamma (j_{\sigma 1}),\Gamma (j_{\sigma 2}),\Gamma (j_{\sigma 3}),....,\Gamma (j_{\sigma p})) \\= & {} \oplus _{r=1}^{p}\acute{r}_{r}\Gamma (j_{\sigma r}) \\= & {} \bigcup \limits _{\begin{array}{c} \phi _{\Gamma (j_{\sigma 1})}\in \vartheta _{_{\Gamma (j_{\sigma 1})}},\phi _{\Gamma (j_{\sigma 2})}\in \vartheta _{\Gamma (j_{\sigma 2})},...,\phi _{\Gamma (j_{\sigma p})}\in \vartheta _{\Gamma (j_{\sigma p})}, \\ \chi _{_{\Gamma (j_{\sigma 1})}}\in \xi _{_{\Gamma (j_{\sigma 1})}},\chi _{\Gamma (j_{\sigma 2})}\in \xi _{\Gamma (j_{\sigma 2})},...,\chi _{\Gamma (j_{\sigma p})}\in \xi _{\Gamma (j_{\sigma p})}, \\ \psi _{_{\Gamma (j_{\sigma 1})}}\in \partial _{_{\Gamma (j_{\sigma 1})}},\psi _{\Gamma (j_{\sigma 2})}\in \partial _{\Gamma (j_{\sigma 2})},...,\psi _{\Gamma (j_{\sigma p})}\in \partial _{_{\Gamma (j_{\sigma p})}} \end{array}}\left\{ \begin{array}{c} \sqrt{1-\Pi _{r=1}^{p}(1-\phi _{\Gamma (j_{\sigma r})}^{2})^{\acute{r}_{r}}}, \\ \Pi _{r=1}^{p}(\chi _{\Gamma (j_{\sigma r})})^{\acute{r}_{r}},\Pi _{r=1}^{p}(\psi _{\Gamma (j_{\sigma r})})^{\acute{r}_{r}} \end{array} \right\} \end{aligned}$$

where $r=1,2,\ldots ,p$, if $\acute{r}$ $=(\acute{r}_{1},\acute{r}_{2}, \acute{r}_{3},..,\acute{r}_{p})^{T}$ denote the weight vector (WV) of $\acute{r}_{r}$ parameters with condition $\acute{r}_{r}\in [0,1]$ with $\Sigma _{r=1}^{p}\acute{r}_{r}=1$ and $\acute{r}_{r}\ge 0.$ Where $(\sigma _{1},\sigma _{2},...,\sigma _{p})$ is the permutation of $\ (1,2,3,...,p)$ such that $m_{\sigma (r-1)}\ge m_{\sigma (r)}$ for all $r=2,3,...,p$.

#### Proof

The proof is similar to above Theorem4.2. $\square$

Moreover, similarity to the SHFSWA operator, the SHFSOWA operator has some important properties, such as idempotency, boundedness, monotonicity.

### Spherical hesitant fuzzy soft hybrid average (SHFSHA) operator

According to Definition 4.1 and 4.4, SHFSWA operators only weight the spherical hesitant fuzzy soft number itself, whereas SHFSOWA operators weight the ordered ranks of the spherical hesitant fuzzy soft  number rather than the arguments themselves. Therefore, in both the SHFSWA and SHFSOWA operators, the weights represent  two distinct aspects. However, merely one of them is taken into account by either operator. In the paragraphs that follow, we’ll suggest using the spherical hesitant fuzzy soft hybrid average (SHFSHA) operator to tackle this issue.

#### Definition 4.6

Suppose $\Gamma (j_{\sigma r})=(\phi _{\Gamma (j_{\sigma r})},\chi _{\Gamma (j_{\sigma r})},\psi _{\Gamma (j_{\sigma r})}),(r=1,2,3,..,p)$ be collecction of SHFSNs $(\Gamma ,J)$ is the mapping defined as $SHFSHA:\Upsilon ^{p}\rightarrow \Upsilon$, where ($\Upsilon$ is the family of all SHFSNs) such that SHFSHA $\Gamma ^{\prime }(j_{\sigma r})=(\phi _{\Gamma ^{\prime }(j_{\sigma r})},\chi _{\Gamma ^{\prime }(j_{\sigma r})},\psi _{\Gamma ^{\prime }(j_{\sigma r})}),(r=1,2,3,..,p).$

$$\begin{aligned} SHFSHA(\Gamma ^{\prime }(j_{\sigma 1}),\Gamma ^{\prime }(j_{\sigma 2}),\Gamma ^{\prime }(j_{\sigma 3}),....,\Gamma ^{\prime }(j_{\sigma p}))=\oplus _{r=1}^{p}\acute{r}_{r}\Gamma ^{\prime }(j_{\sigma r}). \end{aligned}$$

Here WV $\ \acute{r}=(\acute{r}_{1},\acute{r}_{2},\acute{r}_{3},..,\acute{r} _{p})^{T}$ for $\Gamma ^{\prime }(j_{r})$ parameters with $\acute{r}_{r}\in [0,1],$ $\Sigma _{r=1}^{p}\acute{r}_{r}=1$ and $\acute{r}_{r}\ge 0,$ and $\Gamma ^{\prime }(j_{\sigma r})$ is the r-th biggest element of spherical hesitant fuzzy soft arguments $\Gamma ^{\prime }(j_{\sigma r})(\Gamma ^{\prime }(j_{\sigma r})=(n\acute{r}_{r})\Gamma (j_{\sigma r}),r=1,2,3,...,p)$, $\acute{r}=(\acute{r}_{1},\acute{r}_{2},\acute{r} _{3},..,\acute{r}_{p})^{T}$ is the WV of spherical hesitant fuzzy soft arguments $\Gamma (j_{\sigma r})(r=1,2,3,...,p),$ with $\acute{r}_{r}\in [0,1]$, $\Sigma _{r=1}^{p}\acute{r}_{r}=1$ and $\acute{r}_{r}\ge 0,$ and n is the balancing coefficient.

#### Theorem 4.7

Suppose $\Gamma (j_{\sigma r})=(\phi _{\Gamma (j_{\sigma r})},\chi _{\Gamma (j_{\sigma r})},\psi _{\Gamma (j_{\sigma r})}),(r=1,2,3,..,p)$ be collection of SHFSNs $(\Gamma ,J)$ is the mapping defined as $SHFSHA:\Upsilon ^{p}\rightarrow \Upsilon$, where ($\Upsilon$ is the family of all SHFSNs) such that SHFSHA $\Gamma ^{\prime }(j_{\sigma r})=(\phi _{\Gamma ^{\prime }(j_{\sigma r})},\chi _{\Gamma ^{\prime }(j_{\sigma r})},\psi _{\Gamma ^{\prime }(j_{\sigma r})}),(r=1,2,3,..,p).$

$$\begin{aligned} SHFSHA(\Gamma ^{\prime }(j_{\sigma 1}),\Gamma ^{\prime }(j_{\sigma 2}),\Gamma ^{\prime }(j_{\sigma 3}),....,\Gamma ^{\prime }(j_{\sigma p}))=\oplus _{r=1}^{p}\acute{r}_{r}\Gamma ^{\prime }(j_{\sigma r}). \end{aligned}$$

where WV $\acute{r}=(\acute{r}_{1},\acute{r}_{2},\acute{r}_{3},..,\acute{r} _{p})^{T}$ of $\Gamma ^{\prime }(j_{k})$ parameters, with $\acute{r}_{r}\in [0,1]$, $\Sigma _{r=1}^{p}\acute{r}_{r}=1$ and $\acute{r}_{r}\ge 0,$ and $\Gamma ^{\prime }(j_{\sigma r})$ is the k-th biggest element of the spherical hesitant fuzzy soft arguments $\Gamma ^{\prime }(j_{\sigma r})(\Gamma ^{\prime }(j_{\sigma r})=(n\acute{r}_{r})\Gamma (j_{\sigma r}),r=1,2,3,...,p, \acute{r}=(\acute{r}_{1},\acute{r}_{2},\acute{r}_{3},.., \acute{r}_{p})^{T}$ is the WV of spherical hesitant fuzzy soft arguments $\Gamma (j_{\sigma r})(r=1,2,3,...,p),$ with $\acute{r}_{r}\in [0,1]$, $\Sigma _{r=1}^{p}\acute{r}_{r}=1$ and $\acute{r}_{r}\ge 0,$ and n is the balancing coefficient.

$$\begin{aligned} =\bigcup \limits _{\begin{array}{c} \phi _{\Gamma ^{\prime }(j_{\sigma 1})}\in \vartheta _{_{\Gamma ^{\prime }(j_{\sigma 1})}},\phi _{\Gamma ^{\prime }(j_{\sigma 2})}\in \vartheta _{\Gamma ^{\prime }(j_{\sigma 2})},...,\phi _{\Gamma ^{\prime }(j_{\sigma n})}\in \vartheta _{\Gamma ^{\prime }(j_{\sigma n})}, \\ \chi _{_{\Gamma ^{\prime }(j_{\sigma 1})}}\in \xi _{_{\Gamma ^{\prime }(j_{\sigma 1})}},\chi _{\Gamma ^{\prime }(j_{\sigma 2})}\in \xi _{\Gamma ^{\prime }(j_{\sigma 2})},...,\chi _{\Gamma ^{\prime }(j_{\sigma n})}\in \xi _{\Gamma ^{\prime }(j_{\sigma n})}, \\ \psi _{_{\Gamma ^{\prime }(j_{\sigma 1})}}\in \partial _{_{\Gamma ^{\prime }(j_{\sigma 1})}},\psi _{\Gamma ^{\prime }(j_{\sigma 2})}\in \partial _{\Gamma ^{\prime }(j_{\sigma 2})},...,\psi _{_{\Gamma ^{\prime }(j_{\sigma n})}}\in \partial _{_{\Gamma ^{\prime }(j_{\sigma n})}.} \end{array}}\left\{ \begin{array}{c} \sqrt{1-\Pi _{r=1}^{p}(1-\phi _{\Gamma ^{\prime }(j_{\sigma r})}^{2})^{ \acute{r}_{r}}}, \\ \Pi _{r=1}^{p}(\chi _{\Gamma ^{\prime }(j_{\sigma r})})^{\acute{r}_{r}},\Pi _{r=1}^{p}(\psi _{\Gamma ^{\prime }(j_{\sigma r})})^{\acute{r}_{r}} \end{array} \right\} \end{aligned}$$

#### Proof

The proof is directly analogous to above Theorem-4.2. $\square$

Moreover, similarity to the SHFSWA operator, the SHFSHA operator has some important properties, such as idempotency, boundedness, monotonicity.

### Spherical hesitant fuzzy soft weighted geometric aggregation (SHFSWGA) operator

This operator is used to calculate the geometric average of a set of SHFSS. The use of this operator is important because it takes into account both the degree of MG, nMG, NMG, along with the degree of hesitancy of the elements in the SHFSS. This provides a more balanced consideration of all the elements in the set, instead of just focusing on the most dominant ones.

#### Definition 4.8

Suppose $\Gamma (j_{r})=(\phi _{\Gamma (j_{r})},\chi _{\Gamma (j_{r})},\psi _{\Gamma (j_{r})}),(r=1,2,3,..,p)$ be collecction of SHFSNs $(\Gamma ,J)$ having $\acute{r}$ $=(\acute{r}_{1},\acute{r}_{2},\acute{r} _{3},..,\acute{r}_{p})^{T}$ is WV of $\Gamma (j_{r})$ parameters, where $\acute{r}_{r}\in [0,1]$ with $\Sigma _{r=1}^{p}\acute{r}_{r}=1$ and $\acute{r}_{r}\ge 0,$ then SHFSWGA operator is the mapping defined as $SHFSWGA:\Upsilon ^{p}\rightarrow \Upsilon$, where ($\Upsilon$ is the family of all SHFSNs) such that SHFSWGA $\Gamma (j_{r})=(\phi _{\Gamma (j_{r})},\chi _{\Gamma (j_{r})},\psi _{\Gamma (j_{r})}),(r=1,2,3,..,p).$

$$\begin{aligned} SHFSWGA(\Gamma (j_{1}),\Gamma (j_{2}),\Gamma (j_{3}),....,\Gamma (j_{p}))=\prod \limits _{r=1}^{p}(\Gamma (j_{r}))^{\acute{r}_{r}}. \end{aligned}$$

#### Theorem 4.9

Let $\Gamma (j_{r})=(\phi _{\Gamma (j_{r})},\chi _{\Gamma (j_{r})},\psi _{\Gamma (j_{r})}),(r=1,2,3,..,p),$ be an SHFSNs, the aggregated data by SHFSWGA operator is also an SHFSNs, and given by

$$\begin{aligned}{} & {} SHFSWGA(\Gamma (j_{1}),\Gamma (j_{2}),\Gamma (j_{3}),....,\Gamma (j_{p})) \\= & {} \prod \limits _{r=1}^{p}(\Gamma (j_{r}))^{\acute{r}_{r}} \\= & {} \bigcup \limits _{\begin{array}{c} \phi _{\Gamma (j_{1})}\in \vartheta _{\Gamma (j_{1})},\phi _{\Gamma (j_{2})}\in \vartheta _{\Gamma (j_{2})},...,\phi _{\Gamma (j_{p})}\in \vartheta _{\Gamma (j_{p})}, \\ \chi _{\Gamma (j_{1})}\in \xi _{\Gamma (j_{1})},\chi _{\Gamma (j_{2})}\in \xi _{\Gamma (j_{2})},...,\chi _{\Gamma (j_{p})}\in \xi _{\Gamma (j_{p})}, \\ \psi _{\Gamma (j_{1})}\in \partial _{\Gamma (j_{1})},\psi _{\Gamma (j_{2})}\in \partial _{\Gamma (j_{2})},...,\psi _{_{\Gamma (j_{p})}}\in \partial _{_{\Gamma (j_{p})}.} \end{array}}\left\{ \Pi _{r=1}^{p}(\phi _{\Gamma (j_{r})})^{ \acute{r}_{r}},\Pi _{r=1}^{p}(\chi _{\Gamma (j_{r})})^{\acute{r}_{r}},\sqrt{ 1-\Pi _{r=1}^{p}(1-\psi _{\Gamma (j_{r})}^{2})^{\acute{r}_{r}}}\right\} \end{aligned}$$

where $r=1,2,\ldots ,p$, if $\acute{r}$ $=(\acute{r}_{1},\acute{r}_{2}, \acute{r}_{3},..,\acute{r}_{p})^{T}$ denote the WV of $\acute{r}_{r}$ parameters with condition $\acute{r}_{r}\in [0,1]$ with $\Sigma _{r=1}^{p}\acute{r}_{r}=1$ and $\acute{r}_{r}\ge 0.$

#### Proof

This conclusion has to be supported by mathematical induction.

For $$r=2$$;

$$\begin{aligned} SHFSWGA(\Gamma (j_{1}),\Gamma (j_{2}))=(\Gamma (j_{1}))^{\acute{r} _{1}}\otimes (\Gamma (j_{2}))^{\acute{r}_{2}}. \end{aligned}$$

By using the operational law, we have:

$$\begin{aligned} (\Gamma (j_{1}))^{\acute{r}_{1}}= \bigcup \limits _{\phi _{\Gamma (j_{1})}\in \vartheta _{\Gamma (j_{1})},\chi _{\Gamma (j_{1})}\in \xi _{\Gamma (j_{1})},\psi _{\Gamma (j_{1})}\in \partial _{\Gamma (j_{1})}.}\left\{ (\phi _{\Gamma (j_{1})})^{\acute{r} _{1}},(\chi _{\Gamma (j_{1})})^{\acute{r}_{1}},\sqrt{1-(1-\psi _{\Gamma (j_{1})}^{2})^{\acute{r}_{1}}}\right\} . \\ (\Gamma (j_{2}))^{\acute{r}_{2}}= \bigcup \limits _{\phi _{\Gamma (j_{2})}\in \vartheta _{\Gamma (j_{2})},\chi _{\Gamma (j_{2})}\in \xi _{\Gamma (j_{2})},\psi _{\Gamma (j_{2})}\in \partial _{\Gamma (j_{2})}.}\left\{ (\phi _{\Gamma (j_{2})})^{\acute{r} _{2}},(\chi _{\Gamma (j_{2})})^{\acute{r}_{2}},\sqrt{1-(1-\psi _{\Gamma (j_{2})}^{2})^{\acute{r}_{2}}}\right\} . \\ (\Gamma (j_{1}))^{\acute{r}_{1}}\otimes (\Gamma (j_{2}))^{\acute{r}_{2}}= \bigcup \limits _{\begin{array}{c} \phi _{\Gamma (j_{1})}\in \vartheta _{\Gamma (j_{1})},\phi _{\Gamma (j_{2})}\in \vartheta _{\Gamma (j_{2})}, \\ \chi _{\Gamma (j_{1})}\in \xi _{\Gamma (j_{1})},\chi _{\Gamma (j_{2})}\in \xi _{\Gamma (j_{2})}, \\ \psi _{\Gamma (j_{1})}\in \partial _{\Gamma (j_{1})},\psi _{\Gamma (j_{2})}\in \partial _{\Gamma (j_{2})}. \end{array}}\left\{ \begin{array}{c} (\phi _{\Gamma (j_{1})})^{\acute{r}_{1}},(\chi _{\Gamma (j_{1})})^{\acute{r} _{1}},\sqrt{1-(1-\psi _{\Gamma (j_{1})}^{2})^{\acute{r}_{1}}}\oplus \\ (\phi _{\Gamma (j_{2})})^{\acute{r}_{2}},(\chi _{\Gamma (j_{2})})^{\acute{r} _{2}},\sqrt{1-(1-\psi _{\Gamma (j_{2})}^{2})^{\acute{r}_{2}}} \end{array} \right\} . \\ (\Gamma (j_{1}))^{\acute{r}_{1}}\otimes (\Gamma (j_{2}))^{\acute{r}_{2}}= \bigcup \limits _{\begin{array}{c} \phi _{\Gamma (j_{1})}\in \vartheta _{\Gamma (j_{1})},\phi _{\Gamma (j_{2})}\in \vartheta _{\Gamma (j_{2})}, \\ \chi _{\Gamma (j_{1})}\in \xi _{\Gamma (j_{1})},\chi _{\Gamma (j_{2})}\in \xi _{\Gamma (j_{2})}, \\ \psi _{\Gamma (j_{1})}\in \partial _{\Gamma (j_{1})},\psi _{\Gamma (j_{2})}\in \partial _{\Gamma (j_{2})}. \end{array}}\left\{ \begin{array}{c} (\phi _{\Gamma (j_{1})})^{\acute{r}_{1}}(\phi _{\Gamma (j_{2})})^{\acute{r} _{2}},(\chi _{\Gamma (j_{1})})^{\acute{r}_{1}}(\chi _{\Gamma (j_{2})})^{ \acute{r}_{2}}, \\ \sqrt{ \begin{array}{c} (1-(1-\psi _{\Gamma (j_{1})}^{2})^{\acute{r}_{1}})+(1-(1-\psi _{\Gamma (j_{2})}^{2})^{\acute{r}_{2}})- \\ (1-(1-\psi _{\Gamma (j_{1})}^{2})^{\acute{r}_{1}})(1-(1-\psi _{\Gamma (j_{2})}^{2})^{\acute{r}_{2}}) \end{array}} \end{array} \right\} . \\ (\Gamma (j_{1}))^{\acute{r}_{1}}\otimes (\Gamma (j_{2}))^{\acute{r}_{2}}= \bigcup \limits _{\begin{array}{c} \phi _{\Gamma (j_{1})}\in \vartheta _{\Gamma (j_{1})},\phi _{\Gamma (j_{2})}\in \vartheta _{\Gamma (j_{2})}, \\ \chi _{\Gamma (j_{1})}\in \xi _{\Gamma (j_{1})},\chi _{\Gamma (j_{2})}\in \xi _{\Gamma (j_{2})}, \\ \psi _{\Gamma (j_{1})}\in \partial _{\Gamma (j_{1})},\psi _{\Gamma (j_{2})}\in \partial _{\Gamma (j_{2})}. \end{array}}\left\{ \begin{array}{c} (\phi _{\Gamma (j_{1})})^{\acute{r}_{1}}(\phi _{\Gamma (j_{2})})^{\acute{r} _{2}},(\chi _{\Gamma (j_{1})})^{\acute{r}_{1}}(\chi _{\Gamma (j_{2})})^{ \acute{r}_{2}}, \\ \sqrt{1-(1-\psi _{\Gamma (j_{1})}^{2})^{\acute{r}_{1}}(1-\psi _{\Gamma (j_{2})}^{2})^{\acute{r}_{2}}} \end{array} \right\} . \\ (\Gamma (j_{1}))^{\acute{r}_{1}}\otimes (\Gamma (j_{2}))^{\acute{r}_{2}}= \bigcup \limits _{\begin{array}{c} \phi _{\Gamma (j_{1})}\in \vartheta _{\Gamma (j_{1})},\phi _{\Gamma (j_{2})}\in \vartheta _{\Gamma (j_{2})}, \\ \chi _{\Gamma (j_{1})}\in \xi _{\Gamma (j_{1})},\chi _{\Gamma (j_{2})}\in \xi _{\Gamma (j_{2})}, \\ \psi _{\Gamma (j_{1})}\in \partial _{\Gamma (j_{1})},\psi _{\Gamma (j_{2})}\in \partial _{\Gamma (j_{2})}. \end{array}}\left\{ \begin{array}{c} \Pi _{r=1}^{2}(\phi _{\Gamma (j_{r})})^{\acute{r}_{r}},\Pi _{r=1}^{2}(\chi _{\Gamma (j_{r})})^{\acute{r}_{r}}, \\ \sqrt{1-\Pi _{r=1}^{2}(1-\psi _{\Gamma (j_{r})}^{2})^{\acute{r}_{r}}} \end{array} \right\} . \end{aligned}$$

Thus, the results are true for $r=2$. Assume the results also hold for $p=z.$

$$\begin{aligned}{} & {} SHFSWGA(\Gamma (j_{1}),\Gamma (j_{2}),\Gamma (j_{3}),....,\Gamma (j_{z})) \\= & {} \prod \limits _{r=1}^{z}(\Gamma (j_{r}))^{\acute{r}_{r}} \\= & {} \bigcup \limits _{\begin{array}{c} \phi _{\Gamma (j_{1})}\in \vartheta _{\Gamma (j_{1})},\phi _{\Gamma (j_{2})}\in \vartheta _{\Gamma (j_{2})},...,\phi _{\Gamma (j_{z})}\in \vartheta _{\Gamma (j_{z})}, \\ \chi _{\Gamma (j_{1})}\in \xi _{\Gamma (j_{1})},\chi _{\Gamma (j_{2})}\in \xi _{\Gamma (j_{2})},...,\chi _{\Gamma (j_{z})}\in \xi _{\Gamma (j_{z})}, \\ \psi _{\Gamma (j_{1})}\in \partial _{\Gamma (j_{1})},\psi _{\Gamma (j_{2})}\in \partial _{\Gamma (j_{2})},...,\psi _{_{\Gamma (j_{z})}}\in \partial _{_{\Gamma (j_{z})}.} \end{array}}\left\{ \Pi _{r=1}^{z}(\phi _{\Gamma (j_{r})})^{ \acute{r}_{r}},\Pi _{r=1}^{z}(\chi _{\Gamma (j_{r})})^{\acute{r}_{r}},\sqrt{ 1-\Pi _{r=1}^{z}(1-\psi _{\Gamma (j_{r})}^{2})^{\acute{r}_{r}}}\right\} . \end{aligned}$$

Further, suppose that the results are true for $\ p=z+1$, So combined the above two conditions, we have the following form;

$$\begin{aligned} SHFSWGA(\Gamma (j_{1}),\Gamma (j_{2}),\Gamma (j_{3}),...,\Gamma (j_{z}),\Gamma (j_{z+1}))=\otimes _{r=1}^{z}(\Gamma (j_{r}))^{\acute{r} _{r}}+(\Gamma (j_{z+1}))^{\acute{r}_{z+1}}. \\ =\bigcup \limits _{\begin{array}{c} \phi _{\Gamma (j_{1})}\in \vartheta _{\Gamma (j_{1})},\phi _{\Gamma (j_{2})}\in \vartheta _{\Gamma (j_{2})},..., \\ \phi _{\Gamma (j_{z})}\in \vartheta _{\Gamma (j_{z})},\phi _{\Gamma (j_{z+1})}\in \vartheta _{\Gamma (j_{z+1})} \\ \chi _{\Gamma (j_{1})}\in \xi _{\Gamma (j_{1})},\chi _{\Gamma (j_{2})}\in \xi _{\Gamma (j_{2})},..., \\ \chi _{\Gamma (j_{z})}\in \xi _{\Gamma (j_{z})},\chi _{\Gamma (j_{z+1})}\in \xi _{\Gamma (j_{z+1})} \\ \psi _{\Gamma (j_{1})}\in \partial _{\Gamma (j_{1})},\psi _{\Gamma (j_{2})}\in \partial _{\Gamma (j_{2})},..., \\ \psi _{_{\Gamma (j_{z})}}\in \partial _{_{\Gamma (j_{z})}},\psi _{_{\Gamma (j_{z+1})}}\in \partial _{_{\Gamma (j_{z+1})}} \end{array}}\left\{ \begin{array}{c} \Pi _{r=1}^{z}(\phi _{\Gamma (j_{r})})^{\acute{r}_{r}},\Pi _{r=1}^{z}(\chi _{\Gamma (j_{r})})^{\acute{r}_{r}},\sqrt{1-\Pi _{r=1}^{z}(1-\psi _{\Gamma (j_{r})}^{2})^{\acute{r}_{r}}}\oplus \\ (\phi _{\Gamma (j_{z+1})})^{\acute{r}_{z+1}},(\chi _{\Gamma (j_{z+1})})^{ \acute{r}_{z+1}},\sqrt{1-(1-\psi _{\Gamma (j_{z+1})}^{2})^{\acute{r}_{z+1}}} \end{array} \right\} . \\ =\bigcup \limits _{\begin{array}{c} \phi _{\Gamma (j_{1})}\in \vartheta _{\Gamma (j_{1})},\phi _{\Gamma (j_{2})}\in \vartheta _{\Gamma (j_{2})},..., \\ \phi _{\Gamma (j_{z})}\in \vartheta _{\Gamma (j_{z})},\phi _{\Gamma (j_{z+1})}\in \vartheta _{\Gamma (j_{z+1})} \\ \chi _{\Gamma (j_{1})}\in \xi _{\Gamma (j_{1})},\chi _{\Gamma (j_{2})}\in \xi _{\Gamma (j_{2})},..., \\ \chi _{\Gamma (j_{z})}\in \xi _{\Gamma (j_{z})},\chi _{\Gamma (j_{z+1})}\in \xi _{\Gamma (j_{z+1})} \\ \psi _{\Gamma (j_{1})}\in \partial _{\Gamma (j_{1})},\psi _{\Gamma (j_{2})}\in \partial _{\Gamma (j_{2})},..., \\ \psi _{_{\Gamma (j_{z})}}\in \partial _{_{\Gamma (j_{z})}},\psi _{_{\Gamma (j_{z+1})}}\in \partial _{_{\Gamma (j_{z+1})}} \end{array}}\left\{ \begin{array}{c} \Pi _{r=1}^{z}(\phi _{\Gamma (j_{r})})^{\acute{r}_{r}}(\phi _{\Gamma (j_{z+1})})^{\acute{r}_{z+1}},\Pi _{k=1}^{z}(\chi _{\Gamma (j_{r})})^{\acute{ r}_{r}}(\chi _{\Gamma (j_{z+1})})^{\acute{r}_{z+1}}, \\ \sqrt{ \begin{array}{c} (1-\Pi _{k=1}^{z}(1-\psi _{\Gamma (j_{r})})^{\acute{r}_{r}})+(1-(1-\psi _{\Gamma (j_{z+1})}^{2})^{\acute{r}_{z+1}})- \\ (1-\Pi _{k=1}^{z}(1-\psi _{\Gamma (j_{r})})^{\acute{r}_{r}})(1-(1-\psi _{\Gamma (j_{z+1})}^{2})^{\acute{r}_{z+1}}) \end{array}} \end{array} \right\} . \\ =\bigcup \limits _{\begin{array}{c} \phi _{\Gamma (j_{1})}\in \vartheta _{\Gamma (j_{1})},\phi _{\Gamma (j_{2})}\in \vartheta _{\Gamma (j_{2})},..., \\ \phi _{\Gamma (j_{z})}\in \vartheta _{\Gamma (j_{z})},\phi _{\Gamma (j_{z+1})}\in \vartheta _{\Gamma (j_{z+1})} \\ \chi _{\Gamma (j_{1})}\in \xi _{\Gamma (j_{1})},\chi _{\Gamma (j_{2})}\in \xi _{\Gamma (j_{2})},..., \\ \chi _{\Gamma (j_{z})}\in \xi _{\Gamma (j_{z})},\chi _{\Gamma (j_{z+1})}\in \xi _{\Gamma (j_{z+1})} \\ \psi _{\Gamma (j_{1})}\in \partial _{\Gamma (j_{1})},\psi _{\Gamma (j_{2})}\in \partial _{\Gamma (j_{2})},..., \\ \psi _{_{\Gamma (j_{z})}}\in \partial _{_{\Gamma (j_{z})}},\psi _{_{\Gamma (j_{z+1})}}\in \partial _{_{\Gamma (j_{z+1})}} \end{array}}\left\{ \begin{array}{c} \Pi _{r=1}^{z}(\phi _{\Gamma (j_{r})})^{\acute{r}_{r}}(\phi _{\Gamma (j_{z+1})})^{\acute{r}_{z+1}},\Pi _{r=1}^{z}(\chi _{\Gamma (j_{r})})^{\acute{ r}_{r}}(\chi _{\Gamma (j_{z+1})})^{\acute{r}_{z+1}}, \\ \sqrt{(1-\Pi _{r=1}^{z}(1-\psi _{\Gamma (j_{r})}^{2})^{\acute{r}_{r}}(1-\psi _{\Gamma (j_{z+1})}^{2})^{\acute{r}_{z+1}}} \end{array} \right\} . \\ =\bigcup \limits _{\begin{array}{c} \phi _{\Gamma (j_{1})}\in \vartheta _{\Gamma (j_{1})},\phi _{\Gamma (j_{2})}\in \vartheta _{\Gamma (j_{2})},...,\phi _{\Gamma (j_{z})}\in \vartheta _{\Gamma (j_{z})},\phi _{\Gamma (j_{z+1})}^{2}\in \vartheta _{\Gamma (j_{z+1})} \\ \chi _{\Gamma (j_{1})}\in \xi _{\Gamma (j_{1})},\chi _{\Gamma (j_{2})}\in \xi _{\Gamma (j_{2})},...,\chi _{\Gamma (j_{z})}\in \xi _{\Gamma (j_{z})},\chi _{\Gamma (j_{z+1})}\in \xi _{\Gamma (j_{z+1})} \\ \psi _{\Gamma (j_{1})}\in \partial _{\Gamma (j_{1})},\psi _{\Gamma (j_{2})}\in \partial _{\Gamma (j_{2})},...,\psi _{_{\Gamma (j_{z})}}\in \partial _{_{\Gamma (j_{z})}},\psi _{_{\Gamma (j_{z+1})}}\in \partial _{_{\Gamma (j_{z+1})}} \end{array}}\left\{ \begin{array}{c} \Pi _{r=1}^{z+1}(\phi _{\Gamma (j_{r})})^{\acute{r}_{r}},\Pi _{r=1}^{z+1}(\chi _{\Gamma (j_{r})})^{\acute{r}_{r}}, \\ \sqrt{(1-\Pi _{r=1}^{z+1}(1-\psi _{\Gamma (j_{r})}^{2})^{\acute{r}_{r}}}, \end{array} \right\} . \end{aligned}$$

It is obvious from the expression above that aggregated value is also SHFSN. Consequently, the outcome is valid for all n.

$$\begin{aligned} SHFSWGA(\Gamma (j_{1}),...,\Gamma (j_{p}))=\bigcup \limits _{\begin{array}{c} \phi _{\Gamma (j_{1})}\in \vartheta _{\Gamma (j_{1})},\phi _{\Gamma (j_{2})}\in \vartheta _{\Gamma (j_{2})},...,\phi _{\Gamma (j_{p})}\in \vartheta _{\Gamma (j_{p})}, \\ \chi _{\Gamma (j_{1})}\in \xi _{\Gamma (j_{1})},\chi _{\Gamma (j_{2})}\in \xi _{\Gamma (j_{2})},...,\chi _{\Gamma (j_{p})}\in \xi _{\Gamma (j_{p})}, \\ \psi _{\Gamma (j_{1})}\in \partial _{\Gamma (j_{1})},\psi _{\Gamma (j_{2})}\in \partial _{\Gamma (j_{2})},...,\psi _{_{\Gamma (j_{p})}}\in \partial _{_{\Gamma (j_{p})}.} \end{array}}\left\{ \begin{array}{c} \Pi _{r=1}^{p}(\phi _{\Gamma (j_{r})})^{\acute{r}_{r}},\Pi _{r=1}^{p}(\chi _{\Gamma (j_{r})})^{\acute{r}_{r}}, \\ \sqrt{1-\Pi _{r=1}^{p}(1-\psi _{\Gamma (j_{r})}^{2})^{\acute{r}_{r}}} \end{array} \right\} . \end{aligned}$$

which presents the proof. $\square$

#### Property 4.10

Obviously, there are some properties which are usually achieved by SHFSWGA aggregation operators.

(a) Idempotency: let $\Gamma (j_{r})=(\phi _{\Gamma (j_{r})},\chi _{\Gamma (j_{r})},\psi _{\Gamma (j_{r})}),(r=1,2,3,..,p)$ be any collection of SHFSS. If all of the $\Gamma (j_{r})=(\phi _{\Gamma (j_{r})},\chi _{\Gamma (j_{r})},\psi _{\Gamma (j_{r})})$ are identical then there is:

$$\begin{aligned} SHFSWGA(\Gamma (j_{1}),\Gamma (j_{2}),\Gamma (j_{3}),....,\Gamma (j_{p}))=\Gamma (j) \end{aligned}$$

(b) Monotonicity: let $\Gamma ^{\prime }(j_{r})=(\phi _{\Gamma ^{\prime }(j_{r})},\chi _{\Gamma ^{\prime }(j_{r})},\psi _{\Gamma ^{\prime }(j_{r})}),and$ $\Gamma ^{\prime }(j_{r})=(\phi _{\Gamma ^{\prime }(j_{r})},\chi _{\Gamma ^{\prime }(j_{r})},\psi _{\Gamma ^{\prime }(j_{r})})$ where $(r=1,2,3,..,p)$ be any collection of SHFSS. If it satisfied that $\Gamma (j_{r})\le$ $\Gamma ^{\prime }(j_{r})$ for whole $r\in \Upsilon$ then:

$$\begin{aligned} SHFSWGA(\Gamma (j_{1}),\Gamma (j_{2}),\Gamma (j_{3}),....,\Gamma (j_{p}))=SHFSWGA(\Gamma ^{\prime }(j_{1}),\Gamma ^{\prime }(j_{2}),\Gamma ^{\prime }(j_{3}),....,\Gamma ^{\prime }(j_{p})) \end{aligned}$$

(c) Boundedness: let $\Gamma (j_{r})=(\phi _{\Gamma (j_{r})},\chi _{\Gamma (j_{r})},\psi _{\Gamma (j_{r})}),(r=1,2,3,..,p)$ be any collection of SHFSS. Assuming that $\Gamma (j_{r})^{-}=(\min ^{\phi _{\Gamma (j_{r})}},\min ^{\chi _{\Gamma (j_{r})}},\max ^{\psi _{\Gamma (j_{r})}})$ and $\Gamma (j_{r})^{+}=(\max ^{\phi _{\Gamma (j_{r})}},\min ^{\chi _{\Gamma (j_{r})}},\min ^{\psi _{\Gamma (j_{r})}})$ then:

$$\begin{aligned} min\Gamma (j_{r})\le SHFSWGA(\Gamma (j_{1}),\Gamma (j_{2}),\Gamma (j_{3}),....,\Gamma (j_{p}))\le \max \Gamma (j_{r}) \end{aligned}$$

### Spherical hesitant fuzzy soft ordered weighted geometric aggregation (SHFSOWGA) operator

Overall, the SHFSOWGA operator is a useful tool in decision-making problems in the spherical hesitant fuzzy soft environment because it provides a flexible and balanced way to combine the elements in the SHFSS while taking into account the degree of importance of each element.

#### Definition 4.11

Suppose $\Gamma (j_{r})=(\phi _{\Gamma (j_{r})},\chi _{\Gamma (j_{r})},\psi _{\Gamma (j_{r})}),(r=1,2,3,..,p)$ be collecction of SHFSNs $(\Gamma ,J)$ with WV $\acute{r}=(\acute{r}_{1},\acute{r}_{2},\acute{r} _{3},..,\acute{r}_{n})^{T}$ of $\ \Gamma (j_{r})$ parameters, where $\acute{r }_{r}\in [0,1]$ with $\Sigma _{r=1}^{p}\acute{r}_{r}=1$ and $\acute{r} _{r}\ge 0,$ where $(\sigma _{1},\sigma _{2},...,\sigma _{p})$ is the variation (1, 2, 3, ..., *p*) such that $m_{\sigma (r-1)}\ge m_{\sigma (r)}$ for all $r=2,3,...,p$. SHFSOWGA operator is the mapping defined as $SHFSOWGA:\Upsilon ^{p}\rightarrow \Upsilon$, where ($\Upsilon$ is the family of all SHFSNs) such that SHFSOWGA $\Gamma (j_{\sigma r})=(\phi _{\Gamma (j_{\sigma r})},\chi _{\Gamma (j_{\sigma r})},\psi _{\Gamma (j_{\sigma r})}),(r=1,2,3,..,p).$

$$\begin{aligned} SHFSOWGA(\Gamma (j_{\sigma 1}),\Gamma (j_{\sigma 2}),\Gamma (j_{\sigma 3}),....,\Gamma (j_{\sigma p}))=\prod \limits _{r=1}^{p}(\Gamma (j_{\sigma r}))^{\acute{r}_{r}}. \end{aligned}$$

#### Theorem 4.12

Let $\Gamma (j_{\sigma r})=(\phi _{\Gamma (j_{\sigma r})},\chi _{\Gamma (j_{\sigma r})},\psi _{\Gamma (j_{\sigma r})}),(r=1,2,3,..,p),$ be an SHFSNs, the aggregated data by SHFSOWGA operator is also an SHFSNs, and given by

$$\begin{aligned}{} & {} SHFSOWGA(\Gamma (j_{\sigma 1}),\Gamma (j_{\sigma 2}),\Gamma (j_{\sigma 3}),....,\Gamma (j_{\sigma p})) \\= & {} \prod \limits _{r=1}^{p}(\Gamma (j_{\sigma r}))^{\acute{r}_{r}} \\= & {} \bigcup \limits _{\begin{array}{c} \phi _{\Gamma (j_{\sigma 1})}\in \vartheta _{_{\Gamma (j_{\sigma 1})}},\phi _{\Gamma (j_{\sigma 2})}\in \vartheta _{\Gamma (j_{\sigma 2})},...,\phi _{\Gamma (j_{\sigma p})}\in \vartheta _{\Gamma (j_{\sigma p})}, \\ \chi _{_{\Gamma (j_{\sigma 1})}}\in \xi _{_{\Gamma (j_{\sigma 1})}},\chi _{\Gamma (j_{\sigma 2})}\in \xi _{\Gamma (j_{\sigma 2})},...,\chi _{\Gamma (j_{\sigma p})}\in \xi _{\Gamma (j_{\sigma p})}, \\ \psi _{_{\Gamma (j_{\sigma 1})}}\in \partial _{_{\Gamma (j_{\sigma 1})}},\psi _{\Gamma (j_{\sigma 2})}\in \partial _{\Gamma (j_{\sigma 2})},...,\psi _{_{\Gamma (j_{\sigma p})}}\in \partial _{_{\Gamma (j_{\sigma p}).}} \end{array}}\left\{ \begin{array}{c} \Pi _{r=1}^{p}(\phi _{\Gamma (j_{\sigma r})})^{\acute{r}_{r}},\Pi _{r=1}^{p}(\chi _{\Gamma (j_{\sigma r})})^{\acute{r}_{r}}, \\ \sqrt{1-\Pi _{r=1}^{p}(1-\psi _{\Gamma (j_{\sigma r})}^{2})^{\acute{r}_{r}}} \end{array} \right\} \end{aligned}$$

where $r=1,2,\ldots ,p$, if $\acute{r}=\{\acute{r}_{1},\acute{r} _{2},\ldots ,\acute{r}_{p}\}$ denote the weight vector (WV) of $\acute{r} _{r}$ parameters with condition $\acute{r}_{r}\in [0,1]$ with $\Sigma _{r=1}^{p}\acute{r}_{r}=1$ and $\acute{r}_{r}\ge 0.$ Where $(\sigma _{1},\sigma _{2},...,\sigma _{p})$ is the permutation (1, 2, 3, ..., *p*) such that $m_{\sigma (r-1)}\ge m_{\sigma (r)}$ for all $r=2,3,...,p.$

#### Proof

The proof is directly analogous to Theorem-4.9. $\square$

Moreover, similarity to the SHFSWGA operator, the SHFSOWGA operator has some important properties, such as idempotency, boundedness, monotonicity.

### Spherical hesitant fuzzy soft hybrid geometric (SHFSHG) operator

According to Definition 4.8 and 4.11, SHFSWG operators only weight the spherical hesitant fuzzy soft number itself, whereas SHFSOWG operators weight the ordered ranks of the spherical hesitant fuzzy soft  number rather than the arguments themselves. Therefore, in both the  SHFSWG and SHFSOWG operators, the weights depict  two distinct aspects. However, merely one of them is taken into account by either operator. In the paragraphs that follow, we’ll suggest using the spherical hesitant fuzzy soft hybrid geometric (SHFSHG) operator to tackle this issue.

#### Definition 4.13

Suppose $\Gamma (j_{\sigma r})=(\phi _{\Gamma (j_{\sigma r})},\chi _{\Gamma (j_{\sigma r})},\psi _{\Gamma (j_{\sigma r})}),(r=1,2,3,..,p)$ be collecction of SHFSNs $(\Gamma ,J)$ is the mapping defined as $SHFSHG:\Upsilon ^{p}\rightarrow \Upsilon$, where ( $\Upsilon$ is the family of all SHFSNs) such that SHFSHG $\Gamma ^{\prime }(j_{\sigma r})=(\phi _{\Gamma ^{\prime }(j_{\sigma r})},\chi _{\Gamma ^{\prime }(j_{\sigma r})},\psi _{\Gamma ^{\prime }(j_{\sigma r})}),(r=1,2,3,..,p).$

$$\begin{aligned} SHFSHG(\Gamma ^{\prime }(j_{\sigma 1}),\Gamma ^{\prime }(j_{\sigma 2}),\Gamma ^{\prime }(j_{\sigma 3}),....,\Gamma ^{\prime }(j_{\sigma p}))=\prod \limits _{r=1}^{p}\Gamma ^{\prime }(j_{\sigma r})^{\acute{r}_{r}}. \end{aligned}$$

Here WV$\ \acute{r}=(\acute{r}_{1},\acute{r}_{2},\acute{r}_{3},..,\acute{r} _{p})^{T}$ for $\Gamma ^{\prime }(j_{\sigma r})$ parameters, with $\acute{r} _{r}\in [0,1],$ $\Sigma _{r=1}^{p}\acute{r}_{r}=1$ and $\acute{r} _{r}\ge 0,$ and $\Gamma ^{\prime }(j_{\sigma r})$ is the k-th largest element of the spherical hesitant fuzzy soft arguments $\Gamma ^{\prime }(j_{\sigma r})(\Gamma ^{\prime }(j_{\sigma r})=(n\acute{r}_{r})\Gamma (j_{\sigma r}),r=1,2,3,...,p)$, $\acute{r}=(\acute{r}_{1},\acute{r}_{2}, \acute{r}_{3},..,\acute{r}_{p})^{T}$ is the WV of spherical hesitant fuzzy soft arguments $\Gamma (j_{\sigma r})(r=1,2,3,...,p),$ with $\acute{r} _{r}\in [0,1],$ $\Sigma _{r=1}^{p}\acute{r}_{r}=1$ and $\acute{r} _{r}\ge 0,$ and n is the balancing coefficient.

#### Theorem 4.14

Suppose $\Gamma (j_{\sigma r})=(\phi _{\Gamma (j_{\sigma r})},\chi _{\Gamma (j_{\sigma r})},\psi _{\Gamma (j_{\sigma r})}),(r=1,2,3,..,p)$ be collecction of SHFSNs $(\Gamma ,J)$ is the mapping defined as $SHFSHG:\Upsilon ^{p}\rightarrow \Upsilon$, where ($\Upsilon$ is the family of all SHFSNs) such that SHFSHG $\Gamma ^{\prime }(j_{\sigma k})=(\phi _{\Gamma ^{\prime }(j_{\sigma r})},\chi _{\Gamma ^{\prime }(j_{\sigma r})},\psi _{\Gamma ^{\prime }(j_{\sigma r})}),(r=1,2,3,..,p).$

$$\begin{aligned} SHFSHG(\Gamma ^{\prime }(j_{\sigma 1}),\Gamma ^{\prime }(j_{\sigma 2}),\Gamma ^{\prime }(j_{\sigma 3}),....,\Gamma ^{\prime }(j_{\sigma p}))=\prod \limits _{r=1}^{p}\Gamma ^{\prime }(j_{\sigma k})^{\acute{r}_{r}}. \end{aligned}$$

Here WV$\ \acute{r}=(\acute{r}_{1},\acute{r}_{2},\acute{r}_{3},..,\acute{r} _{p})^{T}$ for $\Gamma ^{\prime }(j_{r})$ parameters, with $\acute{r}_{r}\in [0,1],$ $\Sigma _{r=1}^{p}\acute{r}_{r}=1$ and $\acute{r}_{r}\ge 0,$ and $\Gamma ^{\prime }(j_{\sigma r})$ is the r-th biggest element of the spherical hesitant fuzzy soft arguments $\Gamma ^{\prime }(j_{\sigma r})(\Gamma ^{\prime }(j_{\sigma r})=(n\acute{r}_{r})\Gamma (j_{\sigma r}),r=1,2,3,...,p)$, $\acute{r}=(\acute{r}_{1},\acute{r}_{2},\acute{r} _{3},..,\acute{r}_{p})^{T}$ is the WV of spherical hesitant fuzzy soft arguments $\Gamma (j_{\sigma r})(r=1,2,3,...,p),$ with $\acute{r}_{r}\in [0,1],$ $\Sigma _{r=1}^{p}\acute{r}_{r}=1$ and $\acute{r}_{r}\ge 0,$ and n is the balancing coefficient.

$$\begin{aligned} =\bigcup \limits _{\begin{array}{c} \phi _{\Gamma ^{\prime }(j_{\sigma 1})}\in \vartheta _{_{\Gamma ^{\prime }(j_{\sigma 1})}},\phi _{\Gamma ^{\prime }(j_{\sigma 2})}\in \vartheta _{\Gamma ^{\prime }(j_{\sigma 2})},...,\phi _{\Gamma ^{\prime }(j_{\sigma p})}\in \vartheta _{\Gamma ^{\prime }(j_{\sigma p})}, \\ \chi _{_{\Gamma ^{\prime }(j_{\sigma 1})}}\in \xi _{_{\Gamma ^{\prime }(j_{\sigma 1})}},\chi _{\Gamma ^{\prime }(j_{\sigma 2})}\in \xi _{\Gamma ^{\prime }(j_{\sigma 2})},...,\chi _{\Gamma ^{\prime }(j_{\sigma p})}\in \xi _{\Gamma ^{\prime }(j_{\sigma p})}, \\ \psi _{_{\Gamma ^{\prime }(j_{\sigma 1})}}\in \partial _{_{\Gamma ^{\prime }(j_{\sigma 1})}},\psi _{\Gamma ^{\prime }(j_{\sigma 2})}\in \partial _{\Gamma ^{\prime }(j_{\sigma 2})},...,\psi _{_{\Gamma ^{\prime }(j_{\sigma p})}}\in \partial _{_{\Gamma ^{\prime }(jp)}.} \end{array}}\left\{ \begin{array}{c} \Pi _{r=1}^{p}(\phi _{\Gamma ^{\prime }(j_{\sigma r})})^{\acute{r}_{r}},\Pi _{r=1}^{p}(\chi _{\Gamma ^{\prime }(j_{\sigma r})})^{\acute{r}_{r}}, \\ \sqrt{1-\Pi _{r=1}^{p}(1-\psi _{\Gamma ^{\prime }(j_{\sigma r})}^{2})^{ \acute{r}_{r}}} \end{array} \right\} \end{aligned}$$

#### Proof

The proof is directly analogous to Theorem-4.9. $\square$

Moreover, similarity to the SHFSWGA operator, the SHFSHG aggregation operator has some important properties, such as idempotency, boundedness, monotonicity.

## Decision making model under shfs aggregation information

**Figure 1:**
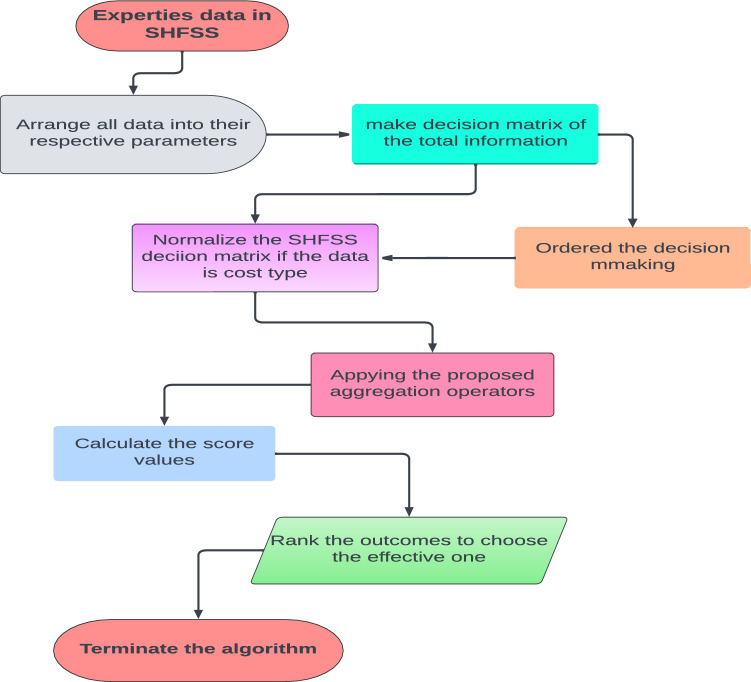
Flow chart.

The flow chart of the the proposed model is shown in Fig. 1.

Here, an MCDM technique for solving MCDM issues that arise in the context of SHFSS is examined, that is based on SHFSWA, SHFSOWA, SHFSWGA, SHFSOWGA, SHFSHG, and SHFSHA aggregation operators.

*Step 1*: Arrange all expert assessment information for every alternative to their respective parameters to create the decision matrix.
*Step 2*: Ordered the overall decision matrix.
*Step 3*: Utilize the SHFSS decision matrix because of the grading,i.e., membership, neutral, and non-membership grade.
*Step 4*: Aggregate SHFSS for each parameter $\hbox {T}\!\!\!\!-$ $=\{f_{1},f_{2},f_{3},...,f_{p}\}$ using the proposed aggregation operators.
*Step 5*: Calculate the score values for each alternative according to the following formula;
  $$\begin{aligned} Score({\hat{J}})=\frac{\left( 1+\frac{1}{x}\sum \limits _{s=1}^{x}\phi _{s}- \frac{1}{y}\sum \limits _{s=1}^{y}\chi _{s}-\frac{1}{z}\sum \limits _{s=1}^{z} \psi _{s}\right) }{2},Score({\hat{J}})\in [0,1]. \end{aligned}$$
Step-6: Rank the outcomes for each alternative $\acute{C} =\{e_{1},e_{2},e_{3},...,e_{m}\}$ and select the most effective one.

### Numerical illustration

Real-world EmDM problems are frequently characterized as being difficult for decision-makers to solve because they are complicated, time-consuming, lack data, and have an impact on mental processes. With the use of membership, neutral, and non-membership values, SHFSS is more adaptable in illustrating the judgment of a group of “decision-makers” in EmDMPs. SHFSS enables decision-makers to choose an unbiased subset of attributes based on their intuition. In order to demonstrate the value of the existing work, we will give a thorough overview of the above-mentioned method to MADM in this part, using an illustrated example

#### Case study 5.1. Supply of emergency aid for post-flooding situation

Natural disasters and global warming exert a serious threat to Pakistan. For years, society has been plagued by catastrophic events like earthquakes, typhoons, flooding, and drought, which frequently destroy the basics on which the existence of huge numbers of families is built. It has been seen that the community’s response determines whether a disaster becomes a catastrophe. Pakistani people face numerous difficulties and require assistance in extreme and devastating weather events, which are becoming more frequent, such as the historic floods of 2022. That puts people’s lives, well-being, and assets in danger. Therefore, the government has established a number of measures in the face of uncontainable natural disasters so that after the disaster hits, the disaster-stricken citizens can be swiftly saved and their lives and production plans can be restored. The use of SHFSS can provide a powerful and flexible tool for modeiling and analyzing emergency supply management information and allowing decision-makers to more effectively manage the complex and uncertain situations that arise in emergency scenarios. Building the emergency shelters is one of them, and it’s very important. According to personal observation, three criteria are typically taken into account when establishing emergency shelters:

- Medication ($${\textbf{f}}_{1}$$),
- Food supplier ($${\textbf{f}}_{2}$$),
- Shelters ($${\textbf{f}}_{3}$$).
  In particular, three kinds of  (alternatives) are taken to provide aid in emergency situations. such as;
- Availability ($${\textbf{e}}_{1}$$),
- Convenience ($${\textbf{e}}_{2}$$),
- Safety ($${\textbf{e}}_{3}$$).

In order to provide Emergency Aid as soon as possible, the emergency command department invited three decision-makers $ER=\{ER_{1},ER_{2},ER_{3}\}$ from government officials, experts in emergency decision-making, experts from international rescue organizations and local residents to participate in the emergency decision-making. These decision-makers evaluated the three alternatives according to three criteria, and have been displayed in Table 1. The three alternatives for emergency aid are listed in detail below.

*Availability* $$\varvec{(e}_{1})$$

Availability refers to the extent to which the supply of emergency aid is accessible and can be obtained in sufficient quantities to meet the needs of those affected by the post-flooding situation. It is the probability that an item will operate competently when it is used to restore emergency conditions in an ideal support environment. The service will be deemed unavailable if the people who have been impacted by the flood are unable to access the service. So, the availability of emergency aid to flood-infected people is most important. In the context of emergency aid, availability can be affected by various factors, such as the location and severity of the flooding, the availability of transportation and communication infrastructure, and the capacity of aid providers.

*Convenience* $$\varvec{(e}_{2})$$

The efficiency of being accessible, simple to use, beneficial, or helpful is known as convenience. Convenience, when compared to the availability of emergency aid due to one’s ease of comfort and suitability becomes effective and also remains during post-flooding periods, which are commonly used for emergency aid. In the context of emergency aid, convenience can be affected by factors such as the accessibility of aid delivery points, the speed of aid delivery, and the suitability of aid for the needs of the recipients. We could save people due to the convenience of emergency aid to the post-flooding areas at the right time.

*Safety* $$\varvec{(e}_{3})$$

The safest zone for the supply of emergency aid is one of the important factors. It refers to the protection of both aid providers and recipients from harm or danger while delivering or receiving emergency aid. It is a condition in which, consequences and situations that can cause damage to physical, psychological, or assets are managed to protect people’s health, property, and well-being. In the context of emergency aid, safety can be affected by various factors, such as the nature and quantity of the aid being delivered, the safety of transportation and communication infrastructure, and the safety of the recipients and aid providers in the affected areas. Safety measures are important to ensure the wellbeing of both aid providers and recipients, as well as the success of the aid delivery process.

**Table 1:** Spherical hesitant fuzzy soft decision matrix.

	$f_{1}$	$f_{2}$	$f_{3}$
$e_{1}$	(0.1)(0.8, 0.3)(0.4, 0.5)	(0.7, 0.35)(0.4)(0.4, 0.1)	(0.4, 0.3)(0.5, 0.25)(0.3)
$e_{2}$	(0.6, 0.3)(0.6)(0.5, 0.45)	(0.5)(0.6, 0.21)(0.35)	(0.25, 0.45)(0.4, 0.15)(0.6, 0.1)
$e_{3}$	(0.5, 0.45)(0.8, 0.4)(0.2)	(0.65)(0.5, 0.2)(0.3, 0.4)	(0.35, 0.2)(0.3)(0.3, 0.1)

Table 1 depicts the spherical hesitant fuzzy soft decision matrix as $\Gamma (j_{r})_{3\times 3}=(\phi _{\Gamma (j_{r})},\chi _{\Gamma (j_{r})},\psi _{\Gamma (j_{r})})_{3\times 3}$. And in this problem, by using the score function, we transformed the Spherical Hesitant fuzzy soft decision matrix to an ordered matrix, presented in Table 2. Let the attribute weight vectors be $\acute{r}=(0.3,0.3,0.4)^{T}$ and for the hybrid matrix we have $\acute{r }=(0.2,0.4,0.4)^{T}$. Using the SHFSWA, SHFSOWA, SHFSWGA, SHFSOWGA, SHFSHA, SHFSHG operators, sequentially. The ranking positions for the alternatives $\acute{C}=\{e_{1},e_{2},e_{3},...,e_{m}\}$ in the decision matrix are listed in Table 2.

**Table 2:** Spherical hesitant fuzzy soft ordered decision matrix.

	$f_{1}$	$f_{2}$	$f_{3}$
$e_{1}$	(0.7, 0.35)(0.4)(0.4, 0.1)	(0.4, 0.3)(0.5, 0.25)(0.3)	(0.1)(0.8, 0.3)(0.4, 0.5)
$e_{2}$	(0.5)(0.6, 0.21)(0.35)	(0.25, 0.45)(0.4, 0.15)(0.6, 0.1)	(0.6, 0.3)(0.6)(0.5, 0.45)
$e_{3}$	(0.65)(0.5, 0.2)(0.3, 0.4)	(0.35, 0.2)(0.3)(0.3, 0.1)	(0.5, 0.45)(0.8, 0.4)(0.2)

now, we apply SHFSWA Operator to find out the aggregated decision values, the outcomes are shown in Table 3a–d.

**Table 3:** The values obtained by SHFSWA Operator for (a): $e_{1}$ (b): $e_{2}$ (c): $e_{3}$ (d): The Score values obtained by SHFSWA Operator.

Weight vector	Membership	Neutral	Non-membership
*a*			
$\{0.3,0.3,0.4\}^{T}$	0.49014836	0.538434644	0.356520492
$\{0.3,0.3,0.4\}^{T}$	0.46424853	0.408057155	0.235215805
$\{0.3,0.3,0.4\}^{T}$	0.32545343	0.401182865	0.381204045
$\{0.3,0.3,0.4\}^{T}$	0.27718389	0.304039757	0.251500877
*b*			
$\{0.3,0.3,0.4\}^{T}$	0.46699314	0.5101698	0.483250058
$\{0.3,0.3,0.4\}^{T}$	0.361940518	0.3446095	0.23599968
$\{0.3,0.3,0.4\}^{T}$	0.516784648	0.3723361	0.468214294
$\{0.3,0.3,0.4\}^{T}$	0.430627463	0.2515056	0.228656824c
*c*			
$\{0.3,0.3,0.4\}^{T}$	0.511483204	0.469317231	0.265640248
$\{0.3,0.3,0.4\}^{T}$	0.484347944	0.356520492	0.171176986
$\{0.3,0.3,0.4\}^{T}$	0.497881544	0.381204045	0.289584624
$\{0.3,0.3,0.4\}^{T}$	0.46942674	0.289584624	0.186606598

Alternatives	Score values		
$e_{1}$	0.3351098		
$e_{2}$	0.3602005		
$e_{3}$	0.4441881		
Ranking order	$e_{3}>e_{2}>e_{1}$		

Now, we apply SHFSWGA Operator to find out the aggregated decision values, the outcomes are shown in Table 4a–d.

**Table 4:** The values obtained by SHFSWGA Operator for (a): $e_{1}$ (b): $e_{2}$ (c): $e_{3}$ (d): The Score values obtained by SHFSWGA Operator.

Weight vector	Membership	Neutral	Non-membership
*a*			
$\{0.3,0.3,0.4\}^{T}$	0.312142862	0.538434644	0.36423959
$\{0.3,0.3,0.4\}^{T}$	0.278213317	0.408057155	0.298073576
$\{0.3,0.3,0.4\}^{T}$	0.253538788	0.401182865	0.402073166
$\{0.3,0.3,0.4\}^{T}$	0.225979433	0.304039757	0.345405666
*b*			
$\{0.3,0.3,0.4\}^{T}$	0.400232526	0.5101698	0.512011791
$\{0.3,0.3,0.4\}^{T}$	0.325089828	0.344609506	0.34855449
$\{0.3,0.3,0.4\}^{T}$	0.506315686	0.372336113	0.498434648
$\{0.3,0.3,0.4\}^{T}$	0.411256129	0.251505604	0.324277365
*c*			
$\{0.3,0.3,0.4\}^{T}$	0.469020933	0.469317231	0.274371646
$\{0.3,0.3,0.4\}^{T}$	0.37495315	0.356520492	0.208762367
$\{0.3,0.3,0.4\}^{T}$	0.454427891	0.381204045	0.311801941
$\{0.3,0.3,0.4\}^{T}$	0.363286918	0.289584624	0.257438284

Alternatives	Score values		
$e_{1}$	0.251046		
$e_{2}$	0.310124		
$e_{3}$	0.389086		
Ranking order	$e_{3}>e_{2}>e_{1}$		

Now, we apply SHFSOWA Operator to find out the aggregated decision values, the outcomes are shown in Table 5a–d.

**Table 5:** The values obtained by SHFSOWA Operator for (a): $e_{1}$ (b): $e_{2}$ (c): $e_{3}$ (d): The Score values obtained by SHFSOWA Operator.

Weight vector	Membership	Neutral	Non-membership
*a*			
$\{0.3,0.3,0.4\}^{T}$	0.477136572	0.564345405	0.366925902
$\{0.3,0.3,0.4\}^{T}$	0.457043512	0.381204045	0.401182865
$\{0.3,0.3,0.4\}^{T}$	0.301841987	0.458390908	0.242080815
$\{0.3,0.3,0.4\}^{T}$	0.262715428	0.309633899	0.264681982
*b*			
$\{0.3,0.3,0.4\}^{T}$	0.497362653	0.531280496	0.474519201
$\{0.3,0.3,0.4\}^{T}$	0.41787512	0.395852373	0.265769671
$\{0.3,0.3,0.4\}^{T}$	0.365491845	0.387743286	0.474519201
$\{0.3,0.3,0.4\}^{T}$	0.531992307	0.288904074	0.265769671
*c*			
$\{0.3,0.3,0.4\}^{T}$	0.522606406	0.517682367	0.2550849
$\{0.3,0.3,0.4\}^{T}$	0.505016454	0.39232987	0.183462951
$\{0.3,0.3,0.4\}^{T}$	0.503245928	0.393261444	0.278077834
$\{0.3,0.3,0.4\}^{T}$	0.484444766	0.298036443	0.2

Alternatives	Score values		
$e_{1}$	0.3137865		
$e_{2}$	0.3410455		
$e_{3}$	0.388486		
Ranking order	$e_{3}>e_{2}>e_{1}$		

Now, we apply SHFSOWGA Operator to find out the aggregated decision values, the outcomes are shown in Table 6a–d.

**Table 6:** The values obtained by SHFSOWGA Operator for (a): $e_{1}$ (b): $e_{2}$ (c): $e_{3}$ (d): The Score values obtained by SHFSOWGA Operator.

Weight vector	Membership	Neutral	Non-membership
*a*			
$\{0.3,0.3,0.4\}^{T}$	0.271736145	0.564345405	0.373610906
$\{0.3,0.3,0.4\}^{T}$	0.249267575	0.381204045	0.421566295
$\{0.3,0.3,0.4\}^{T}$	0.220718335	0.458390908	0.31001912
$\{0.3,0.3,0.4\}^{T}$	0.202468185	0.309633899	0.369014354
*b*			
$\{0.3,0.3,0.4\}^{T}$	0.436851171	0.531280496	0.500360072
$\{0.3,0.3,0.4\}^{T}$	0.394914661	0.395852373	0.35255741
$\{0.3,0.3,0.4\}^{T}$	0.331071278	0.387743286	0.500360072
$\{0.3,0.3,0.4\}^{T}$	0.52109302	0.288904074	0.35255741
*c*			
$\{0.3,0.3,0.4\}^{T}$	0.48605165	0.517682367	0.265179829
$\{0.3,0.3,0.4\}^{T}$	0.465993035	0.39232987	0.215685559
$\{0.3,0.3,0.4\}^{T}$	0.410932989	0.393261444	0.303938585
$\{0.3,0.3,0.4\}^{T}$	0.393974408	0.298036443	0.262951102

Alternatives	Score values		
$e_{1}$	0.219551		
$e_{2}$	0.296789		
$e_{3}$	0.388486		
Ranking order	$e_{3}>e_{2}>e_{1}$		

Now, we find the weighted matrix shown in Table 7 to utilized in hybrid aggregation operators.

**Table 7:** Hybrid Spherical Hesitant Fuzzy Soft Decision matrix.

	$f_{1}$	$f_{2}$	$f_{3}$
$e_{1}$	(0.4, 0.3)(0.5, 0.25)(0.3)	(0.7, 0.35)(0.4)(0.4, 0.1)	(0.1)(0.8, 0.3)(0.4, 0.5)
$e_{2}$	(0.25, 0.45)(0.4, 0.15)(0.6, 0.1)	(0.5)(0.6, 0.21)(0.35)	(0.6, 0.3)(0.6)(0.5, 0.45)
$e_{3}$	(0.35, 0.2)(0.3)(0.3, 0.1)	(0.65)(0.5, 0.2)(0.3, 0.4)	(0.5, 0.45)(0.8, 0.4)(0.2)

Now, we apply SHFSHA Operator to find out the aggregated decision values, the outcomes are shown in Table 8a–d.

**Table 8:** The values obtained by SHFSHA Operator for (a): $e_{1}$ (b): $e_{2}$ (c): $e_{3}$ (d): The Score values obtained by SHFSHA Operator.

Weight vector	Membership	Neutral	Non-membership
*a*			
$\{0.2,0.4,0.4\}^{T}$	0.515026882	0.551891865	0.377635005
$\{0.2,0.4,0.4\}^{T}$	0.295178446	0.480449774	0.412891792
$\{0.2,0.4,0.4\}^{T}$	0.50338435	0.372791927	0.216894354
$\{0.2,0.4,0.4\}^{T}$	0.26907063	0.324534222	0.237144061
*b*			
$\{0.2,0.4,0.4\}^{T}$	0.513787969	0.553264747	0.449619843
$\{0.2,0.4,0.4\}^{T}$	0.53610188	0.45471497	0.314206539
$\{0.2,0.4,0.4\}^{T}$	0.390773189	0.363546708	0.431064713
$\{0.2,0.4,0.4\}^{T}$	0.423878087c	0.298790283	0.301239712
*c*			
$\{0.2,0.4,0.4\}^{T}$	0.550364037	0.544813985	0.2550849
$\{0.2,0.4,0.4\}^{T}$	0.534383168	0.412891792	0.204767251
$\{0.2,0.4,0.4\}^{T}$	0.538757428	0.377635005	0.286193816
$\{0.2,0.4,0.4\}^{T}$	0.522120688	0.286193816	0.229739671

Alternatives	Score values		
$e_{1}$	0.326053413		
$e_{2}$	0.337261701		
$e_{3}$	0.443538136		
Ranking order	$e_{3}>e_{2}>e_{1}$		

Now, we apply SHFSHG Operator to find out the aggregated decision values, the outcomes are shown in Table 9a–d.

**Table 9:** The values obtained by SHFSHG Operator for (a): $e_{1}$ (b): $e_{2}$ (c): $e_{3}$ (d): The Score values obtained by SHFSHG Operator.

Weight vector	Membership	Neutral	Non-membership
*a*			
$\{0.2,0.4,0.4\}^{T}$	0.287376476	0.551891865	0.382680825
$\{0.2,0.4,0.4\}^{T}$	0.217790642	0.372791927	0.429271053
$\{0.2,0.4,0.4\}^{T}$	0.271308542	0.480449774	0.297428117
$\{0.2,0.4,0.4\}^{T}$	0.205613426	0.324534222	0.358973807
*b*			
$\{0.2,0.4,0.4\}^{T}$	0.468205492	0.553264747	0.475734799
$\{0.2,0.4,0.4\}^{T}$	0.526612307	0.45471497	0.394702661
$\{0.2,0.4,0.4\}^{T}$	0.35483341	0.363546708	0.455064951
$\{0.2,0.4,0.4\}^{T}$	0.399097499	0.298790283	0.367146705
*c*			
$\{0.2,0.4,0.4\}^{T}$	0.517090969	0.544813985	0.265179829
$\{0.2,0.4,0.4\}^{T}$	0.495751409	0.412891792	0.233496382
$\{0.2,0.4,0.4\}^{T}$	0.462337738	0.377635005	0.315617669
$\{0.2,0.4,0.4\}^{T}$	0.44325776	0.286193816	0.29036629

Alternatives	Score values		
$e_{1}$	0.223008437		
$e_{2}$	0.298222861		
$e_{3}$	0.399030388		
Ranking order	$e_{3}>e_{2}>e_{1}$		

The comparison between the proposed operators is given below.

**Table 10:** Overall evaluation of the given operators.

Proposed operators	Scoring order
SHFSWA	$e_{3}>e_{2}>e_{1}$
SHFSWGA	$e_{3}>e_{2}>e_{1}$
SHFSOWA	$e_{3}>e_{2}>e_{1}$
SHFSOWGA	$e_{3}>e_{2}>e_{1}$
$\text {SHFSHA}$	$e_{3}>e_{2}>e_{1}$
$\text {SHFSHG}$	$e_{3}>e_{2}>e_{1}$

**Figure 2:**
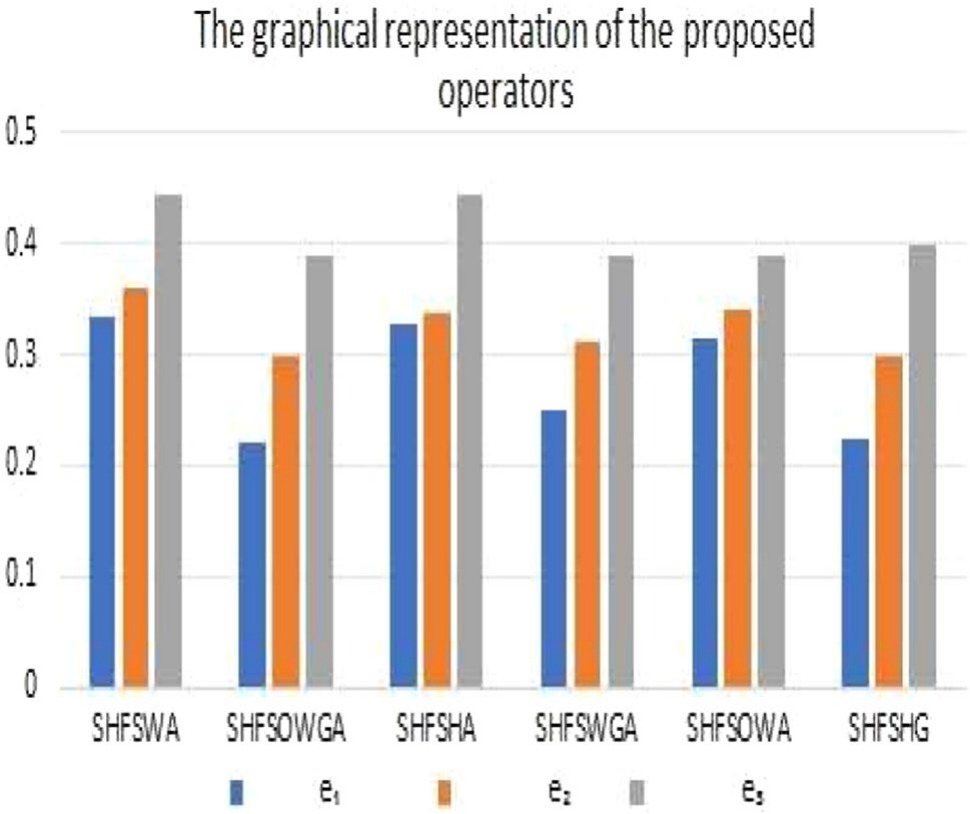
Ranking of alternative.

The graphical representation of alternative ranking is shown in Fig. 2:

Table 10 makes it clear that the overall rating values of the alternatives differ when different operators are used, but the ranking orders of the alternatives are not changed. As a result, the safest alternative is $e_{3}$ .So, the decision makers choose the third alternative with medication, food and safety shelters as emergency aid.

Thus, the proposed MADM technique, which depends on suggested operators, offers an effective alternative to be utilized in decision-support systems.

## Extended EDAS methodology

Suppose $\Gamma (j_{r})=(\phi _{\Gamma (j_{r})},\chi _{\Gamma (j_{r})},\psi _{\Gamma (j_{r})}),(r=1,2,3,..,p)$ be collecction of SHFSNs $(\Gamma ,J),$ $\acute{C}=\{e_{1},e_{2},e_{3},...,e_{m}\}$ be a set of alternative and $F=\{f_{1},f_{2},f_{3},...,f_{p}\},$ as attributes with WVs $\acute{r}=( \acute{r}_{1},\acute{r}_{2},\acute{r}_{3},..,\acute{r}_{p})^{T}$ for $\Gamma (j_{r})$ parameters (attributes), where $\acute{r}_{r}\in [0,1]$ with $\Sigma _{r=1}^{p}\acute{r}_{r}=1$ and $\acute{r}_{r}\ge 0.$ The EDAS algorithm for MADM is developed in the SHFS environment based on the conventional EDAS algorithm. The following are key features:

*Step 1*: Establish the SHFS decision matrix based on the decision maker for each alternative $\acute{C}_{i}$ against their attribute $F_{r}$;
  $$\begin{aligned} X=\left[ \Gamma (j_{ir})\right] _{m\times p}=\left[ \phi _{\Gamma (j_{ir})},\chi _{\Gamma (j_{ir})},\psi _{\Gamma (j_{ir})}\right] _{m\times p}(i=1,2,3,..,m;r=1,2,3,..,p) \end{aligned}$$
*Step 2*: Normalized the aggregated matrix X. As, the data is benefit type so no need to normalize it.
*Step 3*: Determine the value of AvS which is given as
  $$\begin{aligned} AvS= & {} [AvS_{r}]_{1\times p}=\left[ \frac{1}{m}\sum _{i=1}^{m}\Gamma (j_{ir}) \right] _{1\times p} \\= & {} \left[ \bigcup \limits _{\begin{array}{c} \phi _{\Gamma (j_{1r})}\in \vartheta _{\Gamma (j_{1r})},\phi _{\Gamma (j_{2r})}\in \vartheta _{\Gamma (j_{2r})},...,\phi _{\Gamma (j_{mr})}\in \vartheta _{\Gamma (j_{mr})}, \\ \chi _{\Gamma (j_{1r})}\in \xi _{\Gamma (j_{1r})},\chi _{\Gamma (j_{2r})}\in \xi _{\Gamma (j_{2r})},...,\chi _{\Gamma (j_{mr})}\in \xi _{\Gamma (j_{mr})}, \\ \psi _{\Gamma (j_{1r})}\in \partial _{\Gamma (j_{1r})},\psi _{\Gamma (j_{2r})}\in \partial _{\Gamma (j_{2r})},...,\psi _{_{\Gamma (j_{mr})}}\in \partial _{_{\Gamma (j_{mr})}.} \end{array}}\left\{ \begin{array}{c} \sqrt{1-\Pi _{i=1}^{m}(1-\phi _{\Gamma (j_{ir})}^{2})^{1/m}}, \\ \Pi _{i=1}^{m}(\chi _{\Gamma (j_{ir})})^{1/m},\Pi _{i=1}^{m}(\psi _{\Gamma (j_{ir})})^{1/m} \end{array} \right\} \right] _{1\times p} \end{aligned}$$
*Step 4*: Based on computed AvS, determine PDAS and NDAS by utilizing the below formula:
  $$\begin{aligned} PDAS_{ir}=\left[ PDAS_{ir}\right] _{m\times p} \\ =\frac{\max \left( 0,\left[ S(AvS_{ir})-S(AvS_{r})\right] \right) }{ S(AvS_{r})}, \\ NDAS_{ir}=\left[ PDAS_{ir}\right] _{m\times p} \\ =\frac{\max \left( 0,\left[ S(AvS_{r})-S(AvS_{ir})\right] \right) }{ S(AvS_{r})}. \end{aligned}$$
*Step 5*: Further calculate the positive weight distance (SP$_{i}$) and negative weight distance (SN$_{i}$)
  $$\begin{aligned} SP_{i}=\sum _{r=1}^{p}w_{r}PDAS_{ir},\ \ \ \ SN_{i}=\sum _{r=1}^{p}w_{r}NDAS_{ir}. \end{aligned}$$
*Step 6*: Normalized the $SP_{i}$ and $SN_{i}$ by using the below formula:
  $$\begin{aligned} NSP_{i}=\frac{SP_{i}}{\max \left( SP_{i}\right) },\ \ \ \ \ NSN_{i}=1-\frac{ SN_{i}}{\max \left( SN_{i}\right) } \end{aligned}$$
*Step 7*: Compute the appraisal score AS:
  $$\begin{aligned} AS_{i}=\frac{1}{2}(NSP_{i}+NSN_{i}) \end{aligned}$$
*Step 8*: Sort the values in a particular way based on the value of $AS_{i}$ to achieve the superior rank.

**Figure 3:**
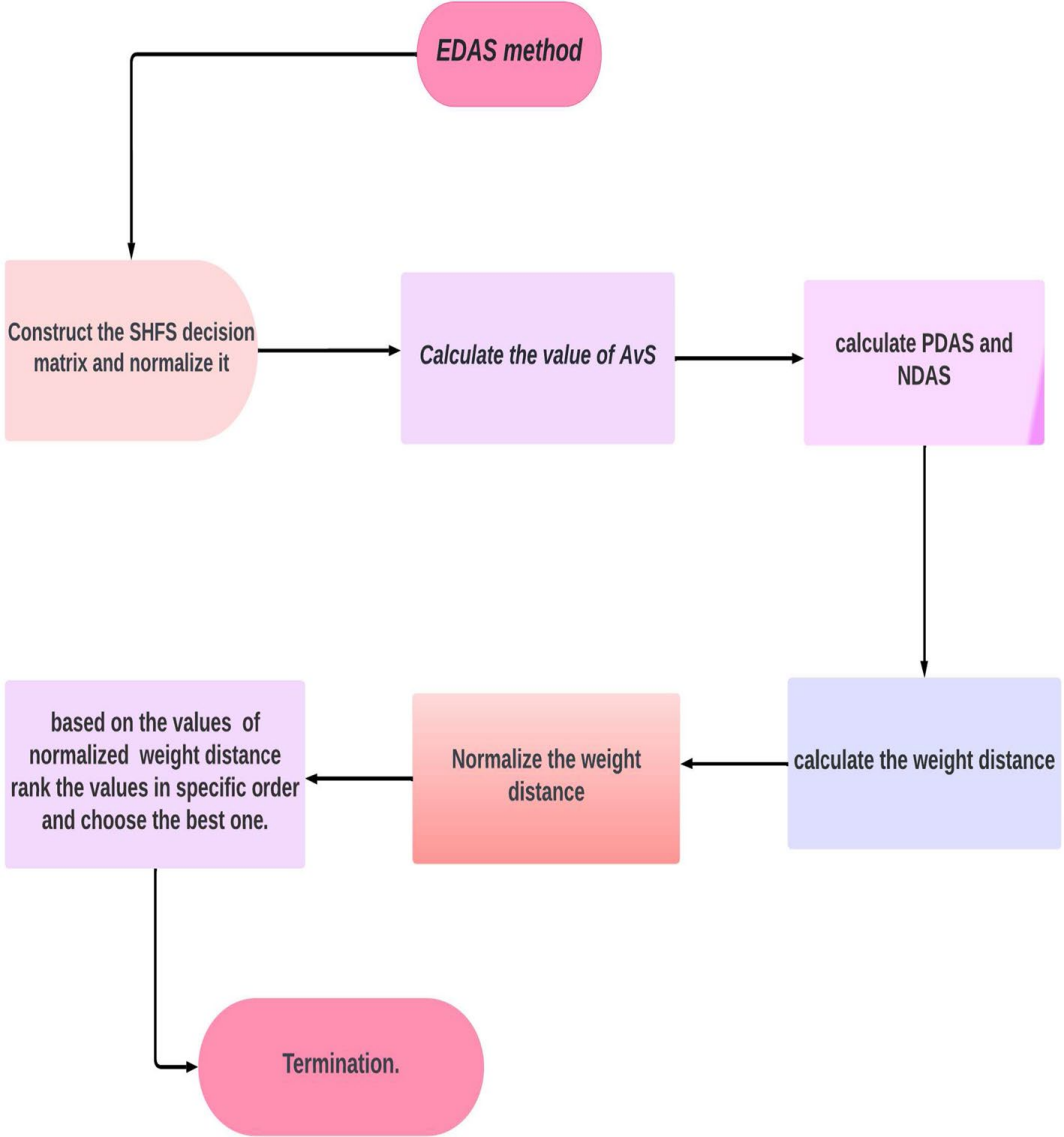
EDAS methodology flow chart.

The flow chart of the EDAS methodology is shown in Fig. 3.

### Illustrative example based on EDAS method

With the same data as previously mentioned in Table 1, we present a real-world MCDM example to demonstrate the effectiveness and supremacy of the analyzed approach. Normalized collective data of experts is given in Table 11 as follows.

**Table 11:** Normalized collective data of experts.

	$f_{1}$	$f_{2}$	$f_{3}$
$e_{1}$	(0.1)(0.8, 0.3)(0.4, 0.5)	(0.7, 0.35)(0.4)(0.4, 0.1)	(0.4, 0.3)(0.5, 0.25)(0.3)
$e_{2}$	(0.6, 0.3)(0.6)(0.5, 0.45)	(0.5)(0.6, 0.21)(0.35)	(0.25, 0.45)(0.4, 0.15)(0.6, 0.1)
$e_{3}$	(0.5, 0.45)(0.8, 0.4)(0.2)	(0.65)(0.5, 0.2)(0.3, 0.4)	(0.35, 0.2)(0.3)(0.3, 0.1)

Now the score value for the normalized collective data of experts is given in Table 12.

**Table 12:** The Score values obtained by weighted Operator.

Attributes	Score values
$f_{1}$	0.244035842
$f_{2}$	0.435787282
$f_{3}$	0.392302065
Ranking Order	$f_{2}>f_{3}>f_{1}$

The results of average solution is given in Table 13:

**Table 13:** The value of average solution $(A_{v}S)$.

	$f_{1}$	$f_{2}$	$f_{3}$
$e_{1}$	0.05	0.4375	0.3375
$e_{2}$	0.1875	0.3725	0.3625
$e_{3}$	0.3375	0.475	0.3875

The PDAS and NDAS value matrix is shown in Table 14.

**Table 14:** PDAS matrix and NDAS matrix.

	$f_{1}$	$f_{2}$	$f_{3}$
PDAS matrix			
$e_{1}$	0	0.00393017	0
$e_{2}$	0	0	0
$e_{3}$	0.382993566	0.089981327	0
NDAS matrix			
$e_{1}$	0.795112064	0	0.139693542
$e_{2}$	0.231670241	0.14522517	0.075967138
$e_{3}$	0	0	0.012240734

The results of $SP_{i}$ & $SN_{i}$ and $NSP_{i}$ & $NSN_{i}$ shown in Tables 15 and 16 respectively.

**Table 15:** The results of $SP_{i}$ and $SN_{i}$.

$SP_{1}= 0.001179051$	$\ SN_{1}= 0.294411036$
$SP_{2}= 0$	$SN_{2}$ $=$ 0.143455478
$SP_{3}= 0.141892468$	$SN_{3}=$ 0.004896293

**Table 16:** The values of $NSP_{i}$ and $NSN_{i}$.

$NSP_{1}= 0.008309468$	$NSN_{1}=\ 0$
$NSP_{2}= 0$	$NSN_{2}= 0.512737429$
$NSP_{3}= 1$	$NSN_{3}= 0.983369192$

Final ranking results are provided in Table 17.

**Table 17:** Appraisal score and Ranking.

$IAS_{i}$	0.004154734	0.256368715	0.991684596
*Ranking* *Order* *of* *EDAS* *method*		$f_{3}>f_{2}>f_{1}$	

**Figure 4:**
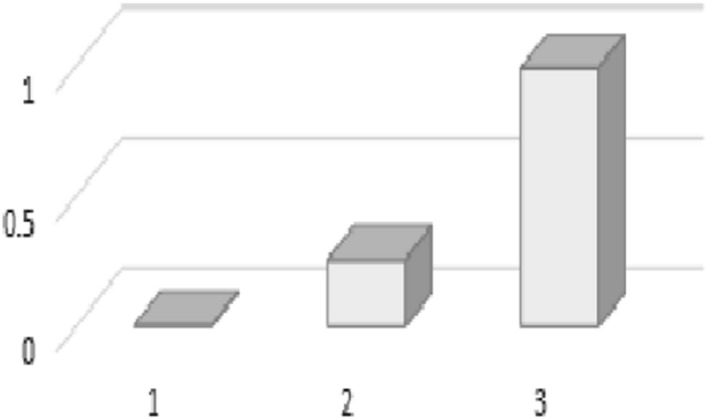
Ranking Result by EDAS Method.

The graphical representation of EDAS method is shown in Fig. 4.

## Comparative analysis

The proposed approaches is better than previously developed decision making techniques. Becaue in it we take into account the hesitant fuzzy sets along with the membership, neutral, and non-membership grades and with the parameterized structure. Firstly, SHFSS provides a more nuanced representation of the decision-making problem by incorporating spherical fuzzy sets. Spherical fuzzy sets allow for more flexibility in modeling the uncertainties and ambiguities of real-world decision-making problems. This can result in more accurate and effective decision-making outcomes. Secondly, SHFSS allows for the integration of both fuzzy sets and soft sets, which can be particularly useful when dealing with decision-making problems that involve both quantitative and qualitative information. The combination of these two approaches can help to balance the strengths and weaknesses of each, leading to more effective decision-making outcomes. Thirdly, the use of hesitant membership functions in SHFSS can help to capture the hesitant attitudes of decision-makers. This can be particularly useful when dealing with decision-making problems that involve multiple decision-makers with differing opinions or preferences. By incorporating hesitant membership functions, SHFSS can help to balance and integrate these different perspectives, leading to more equitable and effective decision-making outcomes. Finally, SHFSS has been shown to be effective in a wide range of applications. This versatility suggests that SHFSS may be a valuable tool in many different decision-making contexts.

**Table:** 

Sets	*MG*	*nMG*	*NMG*	Reliability	Range
Fuzzy set^1^	Yes	No	No	No	$0\le MG\le 1$
IFS^2^	Yes	No	Yes	No	$0\le MG+NMG\le 1$
PFS^15^	Yes	Yes	Yes	No	$0\le MG+nMG+NMG\le 1$
SFS^23^	Yes	Yes	Yes	No	$0\le MG^{2}+nMG^{2}+NMG^{2}\le 1$
SHFSS [Poposed]	Yes	Yes	Yes	Yes	$0\le MG^{2}(\phi _{j})+nMG^{2}(\xi _{j})+NMG^{2}(\partial _{j})\le 1$

## Conclusion

While it is difficult to make definitive claims about the superiority of SHFSS over other decision-making techniques, the approach’s ability to incorporate spherical fuzzy sets, integrate both fuzzy sets and soft sets, capture hesitant attitudes, and its wide range of applications suggest that it may be a valuable tool for decision-makers looking to tackle complex, uncertain problems. So, this study offers the latest numerical modelling of effective management through fuzzy decision support systems. For this purpose, we proposed a hybrid structure of aggregation operators, called spherical hesitant fuzzy soft aggregation operators, to aggregate SHFS data. Following that, we present an algorithm for dealing with SHFS MADM problems. The the numerical illustration along with aggregation operators and EDAS method was provided to validate the established strategy and shows its applicability and efficiency. Therefore, compared to other models currently in use, this new model is more accurate, realistic, and useful. In order to solve the problem of decision-making, this paper aims to establish a customizable soft dicision matrix. According to the study’s findings, the proposed approach is more convenient and consistent with other existing selection processes. We are hopeful that this modified concept will be helpful in dealing with several problems related to uncertainty and will yield more convincing outcomes.

### Limitations of the proposed work

The theory of the aggregation operator and the EDAS approach based on the SHFSS are highly beneficial and dominant in assessing tricky and imprecise information in real-life issues, but they do not operate successfully in specific scenarios or instances due to their structure and requirements. When we came across information in the form of yes, abstain, no, and refusal with an expanded domain, the theories we had developed under the SHFSS information were ignored and could not be processed. In this regard, the sum of square of their data does not belong to the closed interval 0,1. So, here the fundamental criteria violated and we say that these conceptions are also limited.

### Future work

In the future, we will be focusing on developing new operators that improve the accuracy and efficiency of SHFS decision-making methods. This theory can be extended to complex hesitant fuzzy soft sets for gernelized fuzzy set and for Aczel-Alsina aggragation operators. Another challenge in group decision-making with SHFSS is the difficulty of eliciting individual opinions in a consistent and reliable way. The process of assigning hesitancy degrees and determining the spherical shape of the set can be subjective and dependent on individual preferences, which can introduce bias and inconsistency in the decision-making process. To address these challenges, there is a need for further research and standardization in the methods used for group decision-making with SHFSS. This could include the development of standardized aggregation methods and the establishment of best practices for eliciting individual opinions in a consistent and reliable way.

Additionally, some innovative approaches like LINMAP, TAOV for decision-making artificial intelligence and neural networks in the multi-parameter framework of SHFSS would be defined. The theory of yager aggregation operators can be adapted for SHFSS and MADM. Further we needed to create the theory of T-spherical hesitant fuzzy sets and complex T-spherical hesitant fuzzy sets.

### Ethics approval

This article does not contain any studies with human participants or animals performed by any of the authors.

## Data Availability

All data generated or analyzed during this study are included in this article.

## Abbreviations

SHFSS: Spherical hesitant fuzzy soft sets
AO: Aggregation operators
EDAS methodology: Evaluation based on the distance from average solution
DMPs: Decision-making problems
MAGDM: Multiple attribute group decision-making
EmDM: Emergency decision-making
SHFSWA: Spherical hesitant fuzzy soft weighted average
SHFSOWA: Spherical hesitant fuzzy soft ordered weighted average
SHFWGA: Spherical hesitant fuzzy weighted geometric aggregation
SHFSOWG: Spherical hesitant fuzzy soft ordered weighted geometric aggregation
SHFSHA: Spherical hesitant fuzzy soft hybrid average
SHFSHG: Spherical hesitant fuzzy soft hybrid geometric aggregation operator
